# Risk Factors and Comparative Safety of Anti‐PD‐1 Combination Therapies in Advanced Melanoma: A Nationwide Real‐World Cohort Study From China

**DOI:** 10.1002/cam4.71694

**Published:** 2026-04-14

**Authors:** Yuan Qiao, Xiao Fu, Sundus Shukar, Weihong Ge, Wei Yang, Ren bin Jiang, Xiaoyan Dai, Haisheng Chen, Jinyi Zhao, Meng Tang, Fei Mu, Hao Wang, Qiuhui Wu, Xing Xia, Yunan Zhang, Suzhi Ji, Ying Xu, Ruixin Cao, Jiexin Wang, Yu Yao, Jingwen Wang, Yu Fang

**Affiliations:** ^1^ School of Pharmacy Xi'an Jiaotong University Xi'an Shaanxi China; ^2^ Department of Clinical Pharmacy Yan'an University Affiliated Hospital Yan'an Shaanxi China; ^3^ Center for Drug Safety and Policy Research Xi'an Jiaotong University Xi'an Shaanxi China; ^4^ Department of Oncology The First Affiliated Hospital of Xi'an Jiaotong University Xi'an Shaanxi China; ^5^ Department of Pharmacy Nanjing Drum Tower Hospital Nanjing Jiangsu China; ^6^ Department of Pharmacy The Affiliated Cancer Hospital of Zhengzhou University & Henan Cancer Hospital Zheng zhou Henan China; ^7^ Department of Bone and Soft Tissue Tumor and Melanoma The Affiliated Cancer Hospital of Xinjiang Medical University Xinjiang China; ^8^ Department of Pharmacy Gansu Provincial Cancer Hospital lanzhou Gansu China; ^9^ Department of Pharmacy, Shandong Cancer Hospital and Institute Shandong First Medical University & Shandong Academy of Medical Sciences jinan Shandong China; ^10^ Department of Pharmacy, Xijing Hospital Fourth Military Medical University Xi'an Shaanxi China

**Keywords:** anti‐PD‐1, anti‐VEGF, autoimmune disease, combination therapy, immune‐related adverse events, interferon, real‐world

## Abstract

**Backgroud:**

Different anti‐PD‐1 combination therapies are used to improve response rate and combat drug resistance for melanoma in the real world of China. While the reported safety data has remained scarce, especially for the controversial use of interferon. Identification of risk factors and comparative safety of different therapies will be beneficial for physicians to make decisions on rational usage of immunotherapy.

**Methods:**

A nationwide retrospective cohort study consecutively collected advanced melanoma patients treated by anti‐PD‐1 based therapies between July 1, 2018 and June 30, 2023. Univariate and multivariable logistic regression analysis was performed to identify the association between the potential risk factors and the occurrence of immune‐related adverse events (irAEs).

**Results:**

A total of 508 advanced melanoma patients receiving PD‐1 monotherapy (*n* = 181), PD‐1 + tyrosine kinase inhibitors (TKI, *n* = 131), PD‐1 + anti‐vascular endothelial growth factor (anti‐VEGF, *n* = 56), PD‐1 + interferon Alfa‐1b (IFN‐α1b, *n* = 99) and PD‐1 + IFN‐α1b + TKI (*n* = 41) were included. There was no significant difference in irAEs across subtypes of melanoma. While for patients with autoimmune disease of psoriasis and lichen planus, recurrence was observed after immunotherapy. Baseline renal or hepatic dysfunction and prior TKI therapy were risk factors for grade 3–5 irAEs. Multivariate logistic regression model found that compared with PD‐1 monotherapy therapy, PD‐1 based combination therapies had greater toxicity but were tolerated, except PD‐1 + IFN‐α1b + TKI group. Notably, the triple therapy was associated with the highest risk of grade 3–5 irAEs (OR, 3.1; 95% CI: 1.2–8.1; *p* = 0.019), which led to a high rate of hospitalization and permanent treatment discontinuation.

**Conclusion:**

Our results suggested that PD‐1 + TKI, PD‐1 + anti‐VEGF, PD‐1 + IFN‐α1b combination therapies have some adverse events but are generally tolerated sufficiently for continuation of treatment. While the triple therapy PD‐1 + IFN‐α1b + TKI is not recommended because the benefits do not outweigh the risks.

## 1 Introduction

Anti‐programmed cell death 1 (anti‐PD‐1) antibody is a significant breakthrough [1] in treatment of advanced melanoma. However, only 30%–40% [2] patients respond to anti‐PD‐1 monotherapy in cutaneous melanoma (CM). So many clinical trials [3, 4, 5, 6] have explored combinations of anti‐PD‐1 with other agents to improve response rate and combat drug resistance. The most frequently reported was the combination of PD‐1 and cytotoxic T lymphocyte antigen 4 (CTLA‐4) [7, 8]. NCCN [9] and ASCO [10] Guidelines for western and white populations mainly fucus on CM and recommended this combination therapy. But the increased toxicity of Grade 3–5 immune‐related adverse (irAEs) had limited the widespread application of this combination in Asian populations [8, 11].

Since high heterogeneity [12] and poor prognosis [13], it is more urgent to explore other combined therapies for acral (AM) and mucosal (MM) melanoma predominant in Chinese, Japanese, Korean, African and Latin American. Besides BRAF and MEK inhibitors for BRAF‐mutation patients, there are two most widely used types of drugs in Chinese melanoma, anti‐vascular endothelial growth factor (anti‐VEGF) and interferon‐α1b. VEGF mediates immunosuppression in the tumor microenvironment [14] and plays a vital role in tumor metastasis. Anti‐VEGF approved for market use in China includes Tyrosine kinase inhibitors (TKI) [6], bevacizumab [15] and recombinant human endostatin [16]. The combination therapy of anti‐PD‐1 + TKI was recommended in Chinese Society of Clinical Oncology (CSCO) guidelines for MM [17], as the objective response rate (ORR) was raised from 0% ~ 13.3% [18, 19] (anti‐PD‐1 monotherapy) to 24.5%~48.3% [6, 20]. Another type of therapy was the combination of anti‐PD‐1 with interferon‐α1b (anti‐PD‐1 + IFN‐α1b). Over the past decade, preclinical and clinical research have confirmed the essential role of interferons for effective host immunological responses to malignant cells [2, 21]. Although interferon Alfa‐2b (IFN‐α2b) was approved and recommended for adjuvant therapy, its application on advanced melanoma was limited by the significant toxicity [22]. IFN‐α1b, the first genetically engineered drug developed in China, showed lower toxicity than IFN‐α2b [23] and encouraging efficacy in the single center study of metastatic melanoma [24]. Although anti‐PD‐1 combined with IFN‐α1b therapy (PD‐1 + IFN‐α1b) has achieved better therapeutic effects with higher ORR of 32.8%, there are great concerns that combination therapies may also increase the risk of grade 3–5 irAEs. Clinician are also unable to answer patients' safety questions due to the lack of large‐scale in immunotherapy for melanoma experience and high‐level literature evidence.

As the unique mechanism of activating the immune system, anti‐PD‐1 related toxicities can affect multiple organs throughout the whole body [25]. IrAEs can range from mild and moderate to severe and life threatening if not recognized and managed appropriately [26]. Thus, risk assessment of irAEs is extremely important when attempting combination therapy in the real world. Previous studies on irAEs showed that the incidence, spectrum, and severity of irAEs may be related to patient characteristics [27], tumor types [28], combination therapy [29], etc. Comparison safety data between different combination therapy and PD‐1 monotherapy are scarce in AM and MM subtypes, as clinical studies were mostly single‐arm reports with small sample sizes. Hence, this nationwide cohort across different subtypes of melanoma aimed to address the gaps for clinical trials through multi‐center real‐world research. Firstly, we describe the incidence, spectrum, and management of irAEs across different anti‐PD‐1 based combination therapies with a large sample size. Real world research also has advantages of covering patients with comorbidities or autoimmune diseases who were excluded in clinical trials. Secondly, identification of risk factors and management of irAEs can alleviate patients' concerns and optimal management about the toxicity of immunotherapy, not only for AM/MM, but also other solid tumors. Furthermore, comparative safety analysis of different anti‐PD‐1 based combination therapies will be beneficial for physicians to make decisions on rational usage of immunotherapy.

## 2 Method

### 2.1 Patients and Study Design

This nationwide multicenter retrospective cohort study was conducted across 7 institutions in the eastern, western, and central regions of China, which were the tertiary Grade A medical institutions with the largest sample size of melanoma. Patients who were diagnosed as unresectable or metastatic stage III/VI advanced melanoma between July 1, 2018 and June 30, 2023, and receiving at least one dose of anti‐PD‐1 were consecutively included. The subjects treated by anti‐PD‐1 were adult patients (18 years or older) with no severe liver or kidney dysfunction. Patients who received anti‐PD‐1 as adjuvant therapy were excluded. Patients who received experimental treatment combinations with CTLA‐4, cell transfusion therapy, BRAF+/− MEK inhibitor were also excluded.

The study was approved by the Biomedical Ethics Committee of Medical Department, Xi'an Jiaotong University, and obtained exemption from written informed consent. It was registered on the platform of Chinese Clinical Trial Registry (ChiCTR2300077379). The ethics batch number of each center was listed in Table S1.

### 2.2 Data Collection

Standardized electronic case report forms (eCRFs) designed in the Research Electronic Data Capture (REDCap) system were used across all centers, with centralized training provided to ensure uniform data entry. Data were retrospectively collected from each institution's electronic medical record system and Laboratory Information System: sex, age, Eastern Cooperative Oncology Group Performance Status (ECOG PS), disease stage according to the American Joint Committee on Cancer (AJCC) 8th edition, biologic subtypes, number of metastases, presence of brain metastasis or liver metastasis, BRAF mutation status, whether the patient had comorbidities or autoimmune disease, prior therapies, line of therapy, anti‐PD‐1 antibody and different combination therapies, duration of anti‐PD‐1 treatment.

This cohort study followed the Strengthening the Reporting of Observational Studies in Epidemiology (STROBE) reporting guidelines. IrAEs were classified and graded according to the Common Terminology Criteria for Adverse Events (CTCAE) version 5.0. The primary outcomes were the incidence, spectrum, and management of irAEs. The secondary outcomes were the risk factors associated with irAEs of any grade, with particular emphasis on grade 3–5 irAEs.

### 2.3 Statistical Analyses

Continuous variables were described using the median and range. Categorical variables were presented as frequencies and percentages. The baseline characteristics of patients treated with different anti‐PD‐1 based combination therapies were compared using Pearson's chi‐square test or Fisher's exact test. Multicollinearity was assessed by computing the variance inflation factor (VIF). Univariate and multivariable logistic regression analyses were performed to identify the association between the potential risk factors and the occurrence of irAEs. Multivariable analyses were adjusted for age, sex, biological subtypes, combination therapies, and variables with *p* < 0.1 in the univariate analysis. Interaction and stratified analyses were conducted according to sex, age, subtypes of melanoma, number of metastases, anti‐PD‐1 antibody. Missing data BRAF mutation status was not adjusted for as more than 50% of patients didn't undergo testing in the real‐world setting in China. Odds ratios (ORs) with 95% CI were calculated with a significance level of *p* < 0.05 (two sided). All statistical analyses were performed using R statistical software (version 4.2.0) and EmpowerStats software (version 4.2.0).

## 3 Results

### 3.1 Patient Characteristics

**FIGURE 1:**
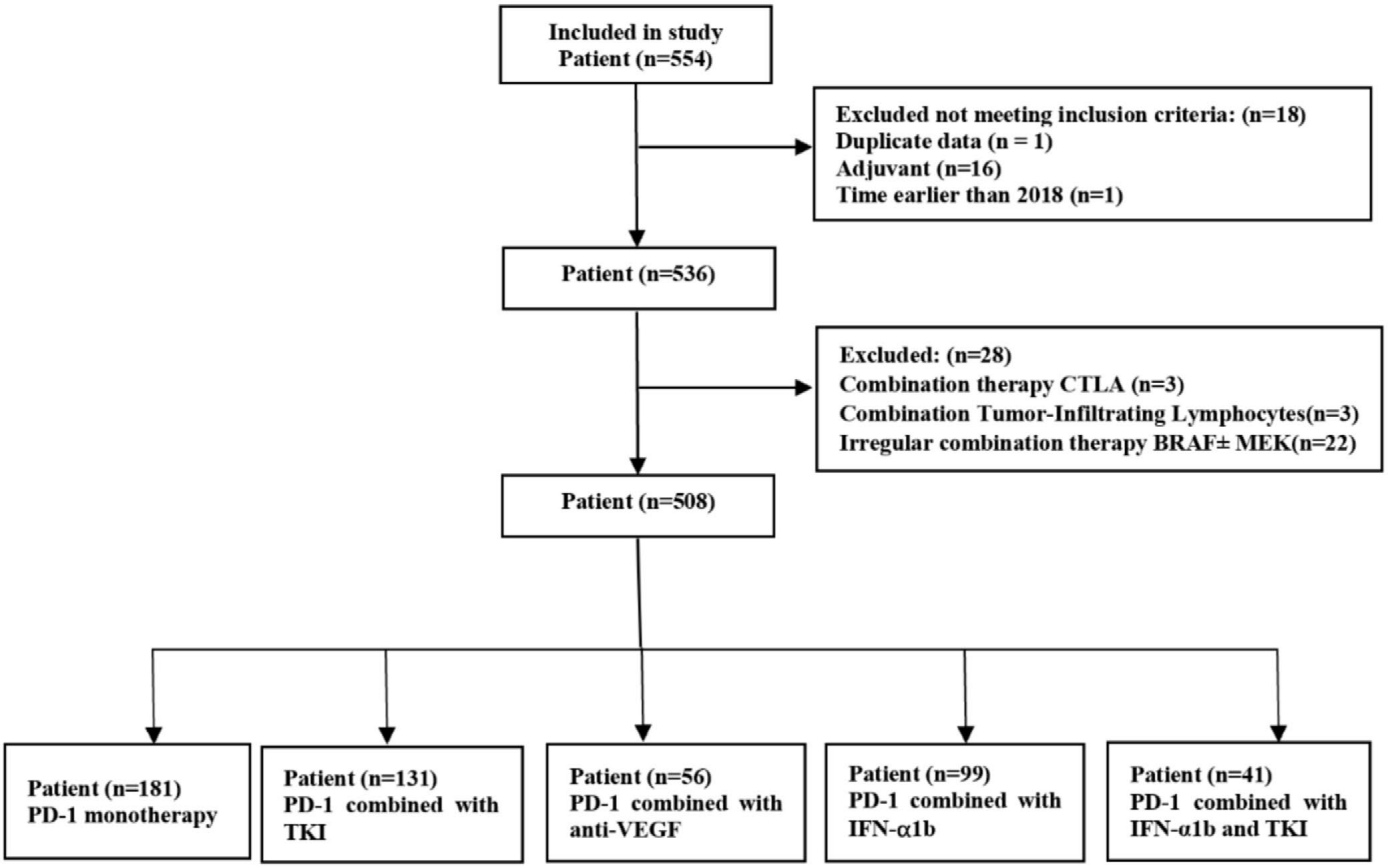
CONSORT flowchart of the study.

Initially, 554 patients were extracted and summarized from RedCap. After applying the inclusion and exclusion criteria, a final study cohort of 508 patients was ultimately included for analysis (Figure 1). Patients' baseline characteristics were listed in Table 1. Our cohort was comprised of 111 (21.9%) non‐acral cutaneous, 194 (38.2%) acral, 158 (31.1%) mucosal, 11 (2.2%) uvea and 34 (6.7%) unknown melanoma patients, respectively. At baseline, there were 245 (48.2%) patients with comorbidities (5 patients with multiple comorbidities), including cardiovascular disease (175, 34.4%), lung disease (25, 4.9%), and renal or hepatic dysfunctions (50, 9.8%). Of these, 347 patients (68.3%) received anti‐PD‐1 based therapies as first line treatment. While there were 332 (65.4%) patients who had received prior chemotherapy, targeted immunotherapy, anti‐VEGF therapy or other systemic treatments, including adjuvant therapies. As shown in Table S2, there were 16 patients with autoimmune diseases treated by PD‐1 based therapies (16, 3.1%), including ankylosing spondylitis, endocrine disorders (adrenocortical hypofunction, thyroiditis) and skin diseases (psoriasis, lichen planus, vitiligo). There were 43 (8.5%) patients treated by anti‐PD‐1 for more than 2 years. More than 50% of patients didn't undergo BRAF mutation testing.

**TABLE 1:** Patient baseline characteristics.

Characteristics	Number of patients (%)						*p*
	Total *n* = 508	PD‐1 *n* = 181	PD‐1 + TKI *n* = 131	PD‐1 + anti‐VEGF *n* = 56	PD‐1 + IFN‐α1b *n* = 99	PD‐1 + TKI + IFN‐α1b *n* = 41	
Sex							
Male	252 (49.6%)	89 (49.2%)	62 (47.3%)	32 (57.1%)	46 (46.5%)	23 (56.1%)	0.620
Female	256 (50.4%)	92 (50.8%)	69 (52.7%)	24 (42.9%)	53 (53.5%)	18 (43.9%)	
Age							
≤ 65	340 (66.9%)	120 (66.3%)	82 (62.6%)	35 (62.5%)	70 (70.7%)	33 (80.5%)	0.224
> 65	168 (33.1%)	61 (33.7%)	49 (37.4%)	21 (37.5%)	29 (29.3%)	8 (19.5%)	
ECOG PS							
0–1	473 (93.1%)	168 (92.8%)	121 (92.4%)	52 (92.9%)	91 (91.9%)	41 (100.0%)	0.482
≥ 2	35 (6.9%)	13 (7.2%)	10 (7.6%)	4 (7.1%)	8 (8.1%)	0 (0.0%)	
Stage							
Unresectable III	63 (12.4%)	25 (13.8%)	25 (19.1%)	4 (7.1%)	8 (8.1%)	1 (2.4%)	0.013
Stage IV	445 (87.6%)	156 (86.2%)	106 (80.9%)	52 (92.9%)	91 (91.9%)	40 (97.6%)	
Subtypes							
Cutaneous	111 (21.9%)	45 (24.9%)	16 (12.2%)	12 (21.4%)	24 (24.2%)	14 (34.1%)	< 0.001
Acral	194 (38.2%)	75 (41.4%)	38 (29.0%)	21 (37.5%)	45 (45.5%)	15 (36.6%)	
Mucosal	158 (31.1%)	39 (21.5%)	69 (52.7%)	20 (35.7%)	21 (21.2%)	9 (22.0%)	
Uvea	11 (2.2%)	3 (1.7%)	2 (1.5%)	1 (1.8%)	4 (4.0%)	1 (2.4%)	
Unknow	34 (6.7%)	19 (10.5%)	6 (4.6%)	2 (3.6%)	5 (5.1%)	2 (4.9%)	
No. of metastases							
< 3	358 (70.5%)	130 (71.8%)	95 (72.5%)	36 (64.3%)	71 (71.7%)	26 (63.4%)	0.643
≥ 3	150 (29.5%)	51 (28.2%)	36 (27.5%)	20 (35.7%)	28 (28.3%)	15 (36.6%)	
Brain metastases							
Yes	57 (11.2%)	19 (10.5%)	18 (13.7%)	10 (17.9%)	9 (9.1%)	1 (2.4%)	0.134
Liver metastasis							
Yes	120 (23.6%)	39 (21.5%)	35 (26.7%)	19 (33.9%)	20 (20.2%)	7 (17.1%)	0.196
BRAF mutation							
Wild‐type	196 (38.6%)	76 (42.0%)	47 (35.9%)	22 (39.3%)	33 (33.3%)	18 (43.9%)	0.500
Mutation	54 (10.6%)	17 (9.4%)	13 (9.9%)	8 (14.3%)	9 (9.1%)	7 (17.1%)	
Not investigated	258 (50.8%)	88 (48.6%)	71 (54.2%)	26 (46.4%)	57 (57.6%)	16 (39.0%)	
Comorbidities	245 (48.2%)	84 (46.4%)	68 (51.9%)	39 (69.6%)	36 (36.4%)	18 (43.9%)	0.002
Ardiovascular	175 (34.4%)	55 (30.4%)	54 (41.2%)	27 (48.2%)	29 (29.2%)	10 (24.4%)	0.020
Lung disease	25 (4.9%)	5 (2.8%)	9 (6.9%)	5 (8.9%)	6 (6.1%)	0 (0.0%)	0.126
Liver/kidney dysfunction	50 (9.8%)	24 (13.3%)	7 (5.3%)	12 (21.4%)	3 (3.0%)	4 (9.8%)	< 0.001
Autoimmune disease							
Yes	16 (3.1%)	1 (1.1%)	1 (0.8%)	1 (1.8%)	9 (9.1%)	3 (7.3%)	< 0.001
Prior therapy	332 (65.4%)	116 (64.1%)	84 (64.1%)	33 (58.9%)	67 (67.7%)	32 (78.0%)	0.356
Prior ICIs	138 (27.2%)	42 (23.2%)	40 (30.5%)	18 (32.1%)	18 (18.2%)	20 (48.8%)	0.002
Prior cytokine	174 (34.3%)	54 (29.8%)	29 (22.1%)	6 (10.7%)	59 (59.6%)	26 (63.4%)	< 0.001
Prior TKI	63 (12.4%)	9 (5.0%)	17 (13.0%)	6 (10.7%)	25 (25.3%)	6 (14.6%)	< 0.001
Prior anti‐VEGF	38 (7.5%)	10 (5.5%)	17 (13.0%)	3 (5.4%)	6 (6.1%)	2 (4.9%)	0.100
Line of therapy							
1 line	347 (68.3%)	126 (69.6%)	80 (61.1%)	37 (66.1%)	77 (77.8%)	27 (65.9%)	0.121
2 line	125 (24.6%)	43 (23.8%)	35 (26.7%)	15 (26.8%)	20 (20.2%)	12 (29.3%)	
≥ 3 line	36 (7.1%)	12 (6.6%)	16 (12.2%)	4 (7.1%)	2 (2.0%)	2 (4.9%)	
Chemotherapy							
No	353 (69.5%)	106 (58.6%)	107 (81.7%)	8 (14.3%)	91 (91.9%)	41 (100.0%)	< 0.001
Yes	155 (30.5%)	75 (41.4%)	24 (18.3%)	48 (85.7%)	8 (8.1%)	0 (0.0%)	
Anti‐PD‐1							
Toripalimab	425 (83.7%)	158 (87.3%)	107 (81.7%)	52 (92.9%)	79 (79.8%)	29 (70.7%)	0.020
Pembrolizumab	83 (16.3%)	23 (12.7%)	24 (18.3%)	4 (7.1%)	20 (20.2%)	12 (29.3%)	
Duration of PD‐1							
0–24 months	465 (91.5%)	163 (90.1%)	121 (92.4%)	53 (94.6%)	88 (88.9%)	40 (97.6%)	0.387
> 24 months	43 (8.5%)	18 (9.9%)	10 (7.6%)	3 (5.4%)	11 (11.1%)	1 (2.4%)	

Abbreviations: Anti‐PD‐1, Anti‐programmed cell death 1; Anti‐VEGF, anti‐angiogenic drug, include bevacizumab and endostar; Cytokine, Include prior interferon (IFN), interleukin (IL) therapy; ECOG PS, Eastern Cooperative Oncology Group performance status; ICIs, Prior Immune checkpoint inhibitors, include prior cytotoxic T lymphocyte antigen 4 (CTLA‐4), Programmed cell death 1 receptor (PD‐1), Programmed cell death‐ligand 1 (PD‐L1); TKI, Tyrosine kinase inhibitors, include Apatinib, Lenvatinib, Axitinib, Anlotinib.

The number of patients treated with PD‐1 monotherapy, PD‐1 + TKI, PD‐1 + anti‐VEGF, PD‐1 + IFN‐α1b, PD‐1 + IFN‐α1b + TKI was 181 (35.6%), 131 (25.8%), 56 (11.0%), 99 (19.5%) and 41 (8.1%), respectively. There were no significant differences in the baseline of sex, age, ECOG PS, number of metastases, whether brain metastases or liver metastases, BRAF mutation status, whether the patient had received prior therapy, line of therapy, and duration of anti‐PD‐1 therapy. Patients with mucosal melanoma were more likely to be treated with PD‐1 + TKI therapy (52.7%), such as anti‐PD‐1 combined with Axitinib. Acral melanoma patients received a higher proportion of PD‐1 combined with interferon (45.5%).

### 3.2 Incidence and Management of irAEs

**FIGURE 2:**
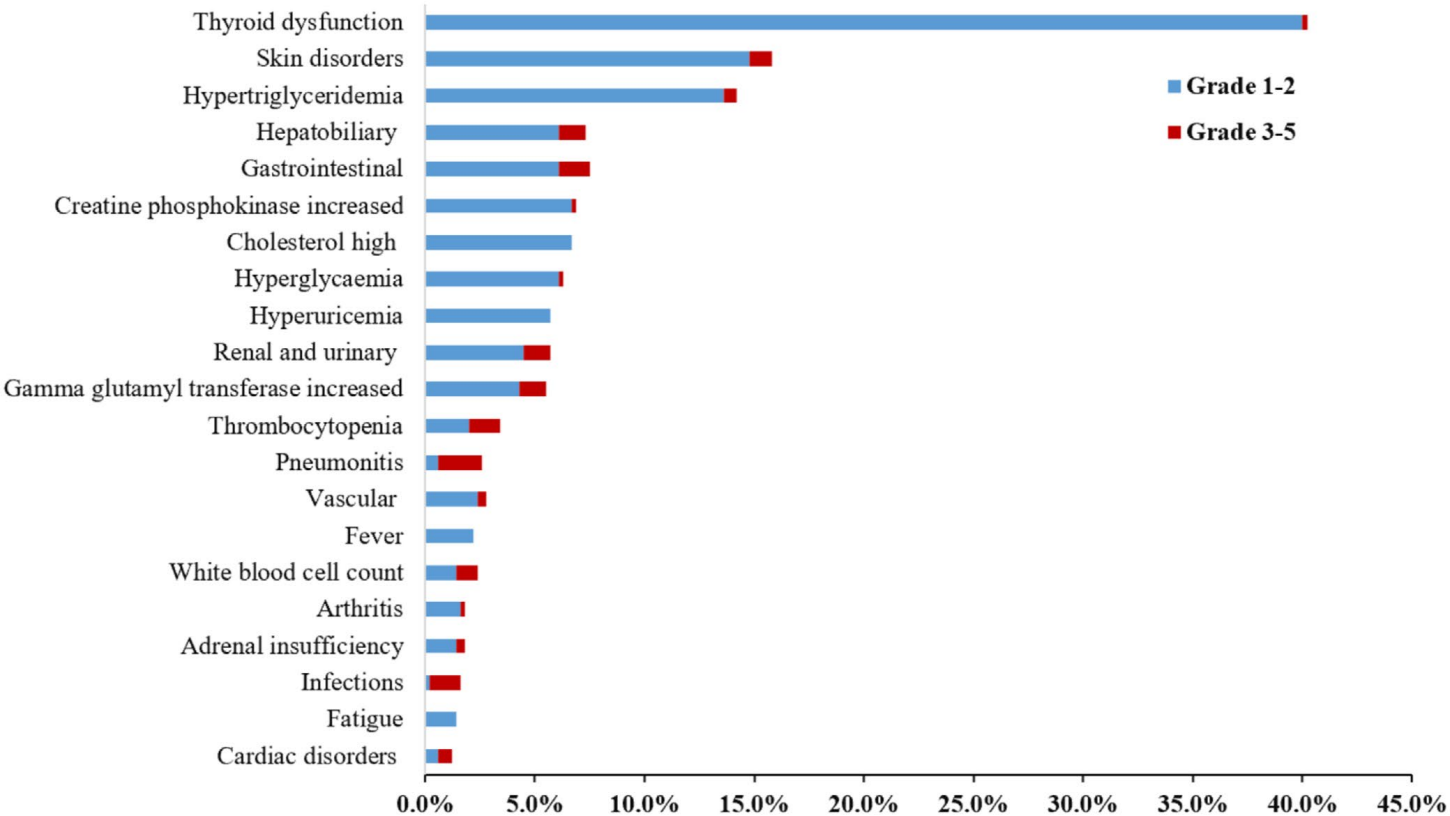
Incidence of irAEs according to category and grade.

Among all 508 patients, 336 (66.1%) developed a total of 775 irAEs. More than two irAEs events occurred in 214 patients, and 56 patients had both mild and severe events. The incidence rate of any grade and grade 3–5 irAEs was 66.1% (322/508) and 13.8% (70/508), respectively. 21 patients required hospitalization and 40 patients required discontinuation; no treatment‐related death occurred, details listed in Tables S3 and S4. As shown in Figure 2, the organ distribution of grade 1–2 irAEs differed from that of grade 3–5. Although thyroid dysfunction and skin toxicity were the most common mild irAEs, the incidence of grade 3–5 events was only 0.2% and 1.0%, respectively. In contrast, the highest incidence of severe irAEs was pneumonitis (10, 2.0%), followed by thrombocytopenia (7, 1.4%). There was no significant difference in the incidence, grades and management of irAEs across different subtypes Table S4.

The management of irAEs was summarized in Tables S4 and S5. The incidence of steroid use, drug discontinuation and hospitalization was 7% (38/508 patients), 8% (40/508 patients) and 4% (21/508 patients), respectively Table S4. Steroids were used to treat 50 irAEs (50/775, 6.5%) in 38 patients (38/508, 7.5%) Table S5. For skin and endocrine irAEs, the incidence of drug discontinuation (2.4% and 6.5%) and hospitalization (0.5% and 5.4%) was relatively low. However, grade 3–5 of hepatobiliary, renal and gastrointestinal disorders often led to discontinuation of PD‐1 and symptomatic treatment. It is worth noting that, although the incidence rate of thrombocytopenia, pneumonitis and cardiac disorders was low, they may lead to a high rate of steroid use, discontinuation of PD‐1, and hospitalization.

### 3.3 Risk Factors for irAEs

**FIGURE 3:**
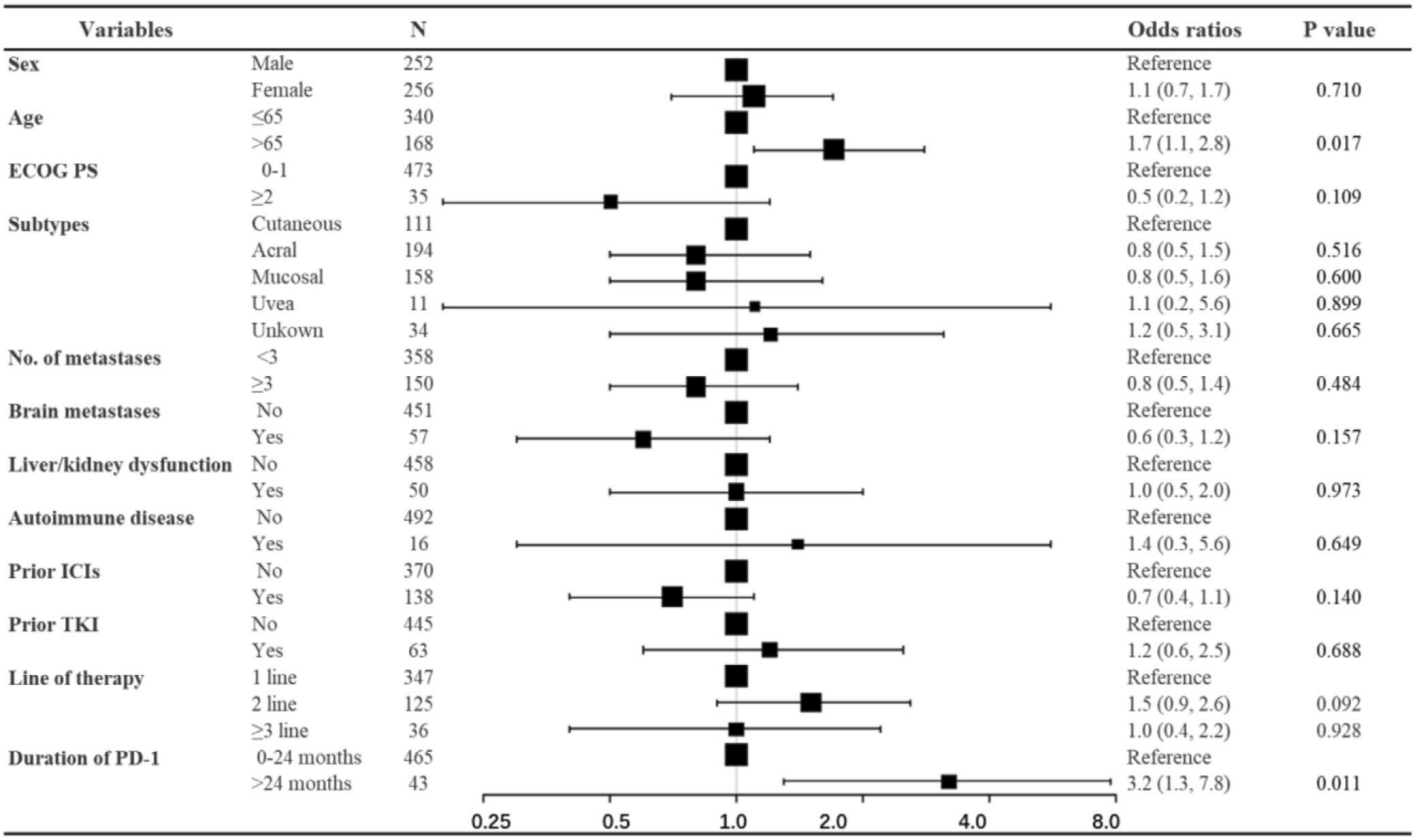
Forest plot of the multivariable logistic regression analysis of potential risk factors of grade 1–2 irAEs.

**FIGURE 4:**
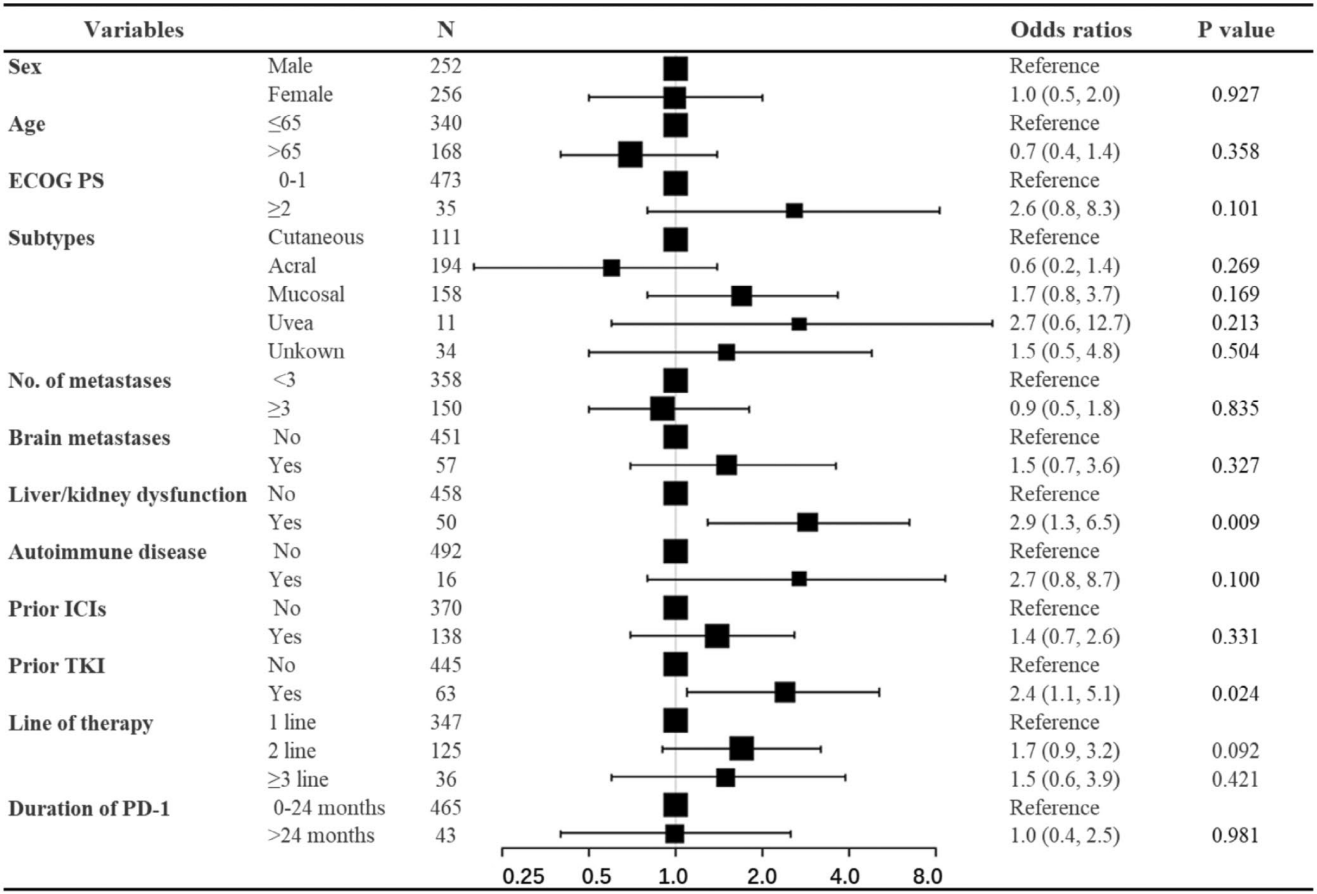
Forest plot of the multivariable logistic regression analysis of potential risk factors of grade 3–5 irAEs.

Multicollinearity testing showed that there was no significant multicollinearity (VIF < 1.5). Univariate and multivariable logistic regression were performed to analyze the association between patient characteristics and irAEs (Table S6 and Figure 3). No significant difference in the incidence of irAEs was found according to biologic subtypes, cardiovascular comorbidities, or prior immunotherapy. In contrast, the incidence of grade 1–2 irAEs was significantly increased in patients older than 65 years or treated by PD‐1 for than 24 months. In addition, baseline renal or hepatic dysfunction and prior TKI therapy were risk factors for grade 3–5 irAEs (Figure 4).

We further analyzed and compared the incidence and organ involvement of irAEs according to different anti‐PD‐1 based therapies. As shown in Table S7, PD‐1 + IFN‐α1b, PD‐1 + IFN‐α1b + TKI significantly increased grade 1–2 skin disorders (36.4% and 26.8%). The incidence of grade 1–2 endocrine disorders was higher in PD‐1 + TKI, PD‐1 + IFN‐α1b, PDc‐1 + IFN‐α1b + TKI groups. In addition, interferon was more likely to cause fever adverse events and required symptomatic treatment with nonsteroidal anti‐inflammatory drugs. There was no significant difference observed in cardiac, respiratory, and musculoskeletal irAEs. Notably, PD‐1 + IFN‐α1b + TKI significantly increased hematologic toxicity, especially in thrombocytopenia. The incidence of any grade and grade 3–5 thrombocytopenia irAEs spiked to 14.6% (PD‐1 monotherapy 2.8%) and 9.8% (PD‐1 monotherapy 0%).

### 3.4 Association Between Different Anti‐PD‐1 Based Combination Therapies and irAEs

As shown in Table 2, Multivariate logistic regression model found that compared with PD‐1 monotherapy therapy, all the anti‐PD‐1 based combination therapies had significantly higher incidence of grade 1–2 irAEs. While only PD‐1 + IFN‐α1b (OR, 2.2; 95% CI: 1.0–4.9; *p* = 0.040), PD‐1 + TKI + IFN‐α1b groups (OR, 3.4; 95% CI: 1.3–8.6; *p* = 0.012) were associated with high incidence of grade 3–5 irAEs in adjusted Modell II. Interestingly, the predictive effect of PD‐1 + IFN‐α1b group on grade 3–5 irAEs change to insignificant after adjustment the variable prior TKI therapy in adjusted Model III (OR, 1.9; 95% CI: 0.9–4.2; *p* = 0.108). But the triple therapy was consistently associated with higher risk of grade 3–5 irAEs (OR, 3.1; 95% CI: 1.2–8.1; *p* = 0.019).

**TABLE 2:** Multivariable Logistic regression analysis of the impact of different anti‐PD‐1 based combination therapy on any grade irAEs and grade 3–5 irAEs.

	*n*	Unadjusted modelI				Adjusted modelII (a)				Adjusted model III (b)			
		Any grade		Grade 3–5		Any grade		Grade 3–5		Any grade		Grade 3–5	
		OR (95% Cl)	*p*	OR (95% Cl)	*p*	OR (95% Cl)	*p*	OR (95% Cl)	*p*	OR (95% Cl)	*p*	OR (95% Cl)	*p*
PD‐1 monotherapy	181	Reference		Reference		Reference		Reference		Reference		Reference	
PD‐1 + TKI	131	1.9 (1.2, 3.0)	0.008	1.5 (0.8, 3.1)	0.232	2.2 (1.3, 3.7)	0.005	1.3 (0.6, 2.9)	0.505	2.2 (1.3, 3.7)	0.005	1.2 (0.6, 2.8)	0.583
PD‐1 + anti‐VEGF	56	1.7 (0.9, 3.1)	0.096	1.2 (0.4, 3.1)	0.770	1.8 (0.9, 3.7)	0.080	0.9 (0.3, 2.6)	0.859	1.8 (0.9, 3.6)	0.084	0.8 (0.3, 2.4)	0.727
PD‐1+ IFN‐α1b	99	3.8 (2.1, 6.6)	< 0.001	2.1 (1.0, 4.4)	0.036	5.1 (2.7, 9.7)	< 0.001	2.2 (1.0, 4.9)	0.040	5.0 (2.6, 9.6)	< 0.001	1.9 (0.9, 4.2)	0.108
PD‐1+ IFN‐α1b + TKI	41	4.9 (2.1, 11.7)	< 0.001	3.5 (1.5, 8.3)	0.004	5.7 (2.2,14.5)	< 0.001	3.4 (1.3, 8.6)	0.012	5.6 (2.2,14.4)	< 0.001	3.1 (1.2, 8.1)	0.019

*Note:* (a) ModelII was adjust for: age, gender, ECOG PS, stage, biologic subtypes, number of metastases, brain metastases, line of therapy, whether chemotherapy was added, anti‐PD‐1, duration of anti‐PD‐1, whether the patient had liver/kidney dysfunction. (b) Model III was adjusted for: age, gender, ECOG PS, stage, biologic subtypes, number of metastases, brain metastases, line of therapy, whether chemotherapy was added, anti‐PD‐1, duration of anti‐PD‐1, whether the patient had liver/kidney dysfunction, prior TKI therapies.

To further confirm that the findings observed are robust to potential confounders, we performed stratified analyses by subgroups defined by major covariables with a sample size greater than 10. The results listed in Table.S8 and Table.S9 revealed a highly consistent pattern.

## 4 Discussion

To the best of our knowledge, this is the first and largest study to compare incidence and spectrum of irAEs among 4 controversial anti‐PD‐1 combination regimens across acral and mucosal subtypes in Asian. The proportion of AM (38.2%) and MM (31.1%) in our cohort was much higher than in western populations [30] (only 2%–3%), consistent with previous epidemiological report [31]. Univariable and multivariable analyses showed there was no significant difference in irAEs across subtypes, indicating anti‐PD‐1 based therapies used in AM/MM won't increase toxicity. Furthermore, for patients who have previously received systemic treatment therapy (line of therapy ≥ 2 line), including ICIs (Prior ICIs), cytokine (Prior cytokine), and anti‐VEGF (Prior anti‐VEGF), immunotherapy can be used again, as the risk of toxicity has not increased. However, the spectrum of irAEs varied in different ethnicities. The most common grade1‐2 and grade 3–4 irAEs in our cohort were thyroid dysfunction/skin and pneumonitis, which was consistent in Japanese patients [32]. While in western populations, the incidence of thyroid dysfunction is not so high, and colitis was the most frequent severe irAEs [33]. Generally, anti‐PD‐1 based therapies were well tolerated in our real‐world research. The usage rate of steroids is lower than reported in clinical trials [34], and no treatment‐related death occurred.

Researchers have shown interest in the potential prognostic relationship between baseline patient characteristics. However, the impact of age on irAEs was controversial in previous reports [35, 36, 37, 38]. The results in our study showed that the incidence of grade 1–2 irAEs was significantly increased in patients over 65 years old. It was consistent with the result of a latest analysis of the Food and Drug Administration Adverse Event Reporting System [37] (*n* = 47,513). The 65–84 age groups had significantly increased risks compared to 18–64, while the > 85 age group showed a decreased risk of irAEs, which indicated that the incidence of irAEs does not uniformly change with age. This may partially explain the reasons for controversial results as most previous studies use a single age [36] or different cut‐off [38] to explore the relationship. As there was only one patient over the age of 85 (1/508) in our cohort, further research verification is needed in the future. In addition, although grade 1–2 irAEs were observed in patients who were older than 65, it showed a downward trend in 3–5 irAEs. Evidence suggested that grade 1–2 irAEs might be predictive of longer survival [39], so current evidence encourages the use of an‐PD1 in elderly melanoma across different combination therapies.

There are relatively few reports on the use of immunotherapy for cancer patients with pre‐existing autoimmune diseases (AID), and existing data was limited. So we listed the details of irAEs and management in AID (Table S2). The results showed manageable toxicity and no significant increase in irAEs, consistent with previous studies on non‐small cell lung cancer and melanoma [40, 41]. But we should notice that for patients with psoriasis and lichen planus, there was a higher risk of recurrence and permanent discontinuation after immunotherapy. In addition, inconsistent with previous literature [42], no flare in ankylosing spondylitis was observed in our study. It may be due to the small sample size (*n* = 2), the shorter duration of anti‐PD‐1, and patients having no active ankylosing spondylitis at baseline. Notably, patients with multiple AID have higher rates of hospitalization. Therefore, for psoriasis, lichen planus, and multiple AID (especially active AID), it is necessary to strengthen clinical monitoring during anti‐PD‐1 therapy.

Although there are many patients with baseline comorbidities in the real world, the safety of immunotherapy for them has not been well explored. Univariable and multivariable analyses showed there was no significant difference in irAEs in patients with cardiovascular disease, indicating anti‐PD‐1 based therapies used in these comorbidities won't increase toxicity. Notably, there was a non‐significant higher incidence of any grade (OR, 3.1; 95% CI: 1.0–9.8; *p* = 0.059) and grade 3–5 irAEs (OR, 2.9; 95% CI: 1.0–8.4; *p* = 0.052) in patients with lung disease. So, it should be cautious when using immunotherapy in these patients, especially for the irAEs of pneumonia. We found only one article about the safety and efficacy of anti‐PD‐1 in patients with baseline cardiac, renal, or hepatic dysfunction [43]. The results showed that patients with baseline comorbidities demonstrated encouraging PFS and OS after immunotherapy, and without remarkable exacerbation of baseline organ dysfunction. However, in our research, multivariable analysis showed that baseline renal or hepatic dysfunction was a risk factor for grade 3–5 irAEs. But most of them were worsening of baseline comorbidities rather than newly developed organs. We speculate that the possible reason may be that patients in previous studies were treated by monotherapy [43]. Further research with larger samples was needed on the PD‐1 based combination therapy for patients with baseline renal or hepatic dysfunction.

Different anti‐PD‐1 based combination therapies are strong risk factors for incidence and spectrum of irAEs. Generally, anti‐PD‐1 based combination therapies had greater toxicity than anti‐PD‐1 monotherapy, but were tolerable in melanoma patients, except PD‐1 + IFN‐α1b + TKI group. Evidence suggested that low grades irAEs might be predictive of better efficacy [39, 44, 45]. This association may be due to the antigen cross‐presentation, so that immunotherapy may lead to activated T cells between tumors and normal tissues which shared antigens [46]. Therefore, the higher incidence of grade 1–2 irAEs caused by combined regimens may indicate a better prognosis. Thus, patients with grade 1–2 irAEs should be advised to improve medication compliance without discontinuation of immunotherapy. In our cohort, there were 131 patients treated by PD‐1 combined with TKI, mainly comprised of Apatinib, Lenvatinib, Axitinib, Anlotinib. Adjusted multivariate analysis revealed that PD‐1 + TKI group had a higher risk of irAEs, but this wasn't significant in grade 3–5 irAEs. Clinicians should pay attention to hypertensive adverse events and hepatorenal toxicity when applying this combination therapy. However, this TKI combination therapy did not significantly increase serious adverse events. Another type of anti‐VEGF bevacizumab demonstrated acceptable safety. Studies showed that VEGF might immunosuppress the tumor microenvironment [47] and improve responses to immune checkpoint therapy. PD‐1 + anti‐VEGF has showed promising in mucosal melanoma and other solid tumors [6, 48, 49]. In the MM subtype analysis, compared with anti‐PD‐1 monotherapy, PD‐1 + TKI showed significant higher ORR (48.3% vs. 0%) and PFS (7.5 months vs. 3.3 months) in the clinical trials [6, 18]. PD‐1 + Bevacizumab demonstrated substantial activity in patients with untreated melanoma brain metastasis with higher ORR (54.1%), and promising OS (4.3 years) [50]. Based on the real‐world treatment results of each center, the treatment selection of PD‐1 + anti‐VEGF in MM among different centers was consistent with guidelines and literature recommendations. The safety data in our study further supports the use of PD‐1 plus VEGF inhibitor in not only MM, but also untreated melanoma brain metastasis.

The study showed that PD‐1 + IFN‐α1b increased the incidence of any grade and grade 3–5 irAEs. Nevertheless, considering the specific types of irAEs caused by it, thyroid dysfunction, skin toxicity, hypertriglyceridemia and fever were not life‐threatening. What's more, thyroid dysfunction and skin toxicity were reported to be associated with better prognosis [51]. And the discontinuation rate and incidence of serious irAEs were all lower than the previously reported PD + CTLA‐4 combination therapy [52]. In addition, although PD‐1 + IFN‐α2b wasn't recommended in CSCO guidelines due to potential toxicity, Professor Gao Tianwen's [24, 53, 54] team from Xijing Hospital, Fourth Military Medical University, explores the use of PD‐1 + IFN‐α1b as a main treatment option. And literature reported that PD‐1 + IFN‐α1b demonstrated an improved OS benefit (17.3 months, 95% Cl: 12.4–22.2 months) [24] than anti‐PD‐1 + CTLA (7.6 ~ 15.6 months) [54, 55] in AM subtypes. PD‐1 + IFN‐α1b may potentially increase the immunogenicity of AM, as Type I IFN plays an important role in the generation and activity of cytotoxic T‐lymphocytes, and activate CD4^+^, CD8^+^ T cells [2]. Thus, the acceptable toxicity in 99 rare melanoma patients treated by PD‐1 + IFN‐α1b provided possibilities for future attempts at this combination therapy. Interestingly, multivariate regression analysis indicated that prior TKI therapy was a risk factor for grade 3–5 irAEs. And the predictive effect of PD‐1 + IFN‐α1b group on grade 3–5 irAEs changed to insignificant after adjusting the variable prior TKI therapy. Previous studies had reported that sequential PD‐1 and EGFR‐TKI Osimertinib may increase toxicity in non‐small‐cell lung cancer [29]. Recent research identified IFN‐expression is enriched by numerous VEGFR TKIs, as increasing senescence marker expression and promoting type I IFN and STING signaling [56]. Type I IFN plays an important role in the generation and activity of cytotoxic T‐lymphocytes, and activate CD4^+^, CD8^+^ T cells [2]. IFN‐α are pleiotropic cytokines belonging to type I IFN [57], so we assumed that the reason for the higher grade 3–5 irAEs risk when prior TKI therapy was used before PD‐1 + IFN‐α1b may be due to synergistic activation.

Similarly, the triple therapy with PD‐1 + IFN‐α1b + TKI in our research significantly increased grade 3–5 irAEs, especially in hematologic toxicity, which led to a high hospitalization and permanent discontinuation rate. Previous studies had shown that anti‐PD‐1 monotherapy had a low incidence of causing hematologic mortality and irAEs, while the combination with chemotherapy might magnify the toxicity [58]. We have collected information about whether chemotherapy was used in each therapy group. There was no chemotherapy added in this triple therapy. There was currently one report on the triple therapy and the incidence of grade 3–5 irAEs was 29.1% (*n* = 55). Although no severe hematologic toxicity was reported in this single‐arm study, the incidence of grade 3–5 irAEs caused by PD‐1 + IFN‐α1b + TKI was much higher than the previous report of PD‐1 + IFN‐α1b [24] (8.6%). Although the mechanisms to explain synergistic toxicity are still not clear, the clinical importance of drug safety prompts us to report these findings to facilitate the awareness of the potential toxicity of this combination. Notably, the triple therapy hasn't shown superior efficacy, the reported ORR, PFS and OS was 9.1%, 2.8 months and 17.6 months. Thus, we think that the triple therapy was not recommended as the current evidence suggesting that the risk outweighs the benefit. Further larger studies will be needed to more definitively determine the risk and benefit of the triple therapy.

There are some limitations in our study. First, the study was retrospective and there might be unmeasured confounding and bias. To minimize possible bias, we consecutively extracted the data from Redcap system, then proceeded strictly according to the inclusion and exclusion criteria. However, recall bias exists as the incomplete data in a retrospective study and some features were recorded after the event. Confounding is possible in this study because the allocation of treatment was probably determined by clinical factors that we are not fully aware of. Second, the incidence of grade 1–2 irAEs may be underestimated as we only reported irAEs that were clearly determined by medical records or clinical physicians. The adverse events most likely caused by others, such as nausea, vomiting, and blood toxicity induced by chemotherapy, were not recorded. However, the comparison results between different groups may not be affected because the standards between each group are unified. Third, although our study is currently the largest melanoma cohort study in Asia, the sample size of some subgroups, such as PD‐1 + IFN‐α1b + TKI groups or certain specific irAEs, was still insufficient. Further research with a larger cohort was needed in the future.

## 5 Conclusions

Anti‐PD‐1 based combination therapies had greater toxicity than anti‐PD‐1 monotherapy, but were tolerable in Asian melanoma across biologic subtypes, including patients with comorbidities or non‐active autoimmune disease. Patients with grade 1‐2 irAEs should be advised to improve medication compliance without discontinuation of immunotherapy. The incidence of grade 1–2 irAEs was higher in patients who were over 65 years old and treated with anti‐PD‐1 for more than 2 years. Baseline renal or hepatic dysfunction, lung disease and prior TKI therapy were risk factors for grade 3–5 irAEs. So, it should be cautious and strengthen monitoring when using immunotherapy in these patients. The incidence, grade and spectrum of irAEs were associated with different anti‐PD‐1 based combination therapies. Multivariable adjusted regression analysis found that compared with PD‐1 monotherapy, the toxicity of PD‐1 + TKI, PD‐1 + anti‐VEGF, PD‐1 + IFN‐α1b therapies was acceptable. The safety data in our study further support the use of them in MM subtype, untreated melanoma brain metastasis and AM subtype respectively. While PD‐1 + IFN + α1b + TKI treatment was associated with the highest incidence of grade 3–5 irAEs, which led to a high hospitalization and permanent discontinuation rate. The triple therapy was not recommended as the current evidence suggests that the risk outweighs the benefit.

## Author Contributions

Yuan Qiao and Xiao Fu contributed to the design, data acquisition, and quality control of the study. Yuan Qiao wrote the manuscript, Sundus Shukar revised the manuscript, and helped improve the English quality. Yu Fang, Jingwen Wang, Yu Yao, Weihong Ge, Wei Yang, Renbing Jang, Xiaoyan Dai, and Haishen Chen supervised the whole project and provided important and critical support. Yu Fang was responsible for the conception and design of the project, fund acquisition, and manuscript revision. Jinyi Zhao, Meng Tang, Fei Mu, Hao Wang, Qiuhui Wu, Xing Xia, Yunnan Zhang, Suzhi Ji, Ying Xu, Ruixin Cao, and Jiexin Wang collected clinical data. All authors contributed to the research.

## Funding

This work was supported by the Key Research and Development Program of the Ministry of Science and Technology—Northwest Region Natural Population Cohort Research Project (Grants 2017YFC0907200 and 2017YFC0907201), the National Natural Science Foundation of China (Grant No. 82103944) and the Shaanxi Provincial Natural Science Foundation (Grant No. 2020JQ‐090).

## Ethics Statement

This nationwide multi‐institutional retrospective cohort study was approved by the Biomedical Ethics Committee of the Medical Department, Xi'an Jiaotong University, and was registered on the platform of the Chinese Clinical Trial Registry (ChiCTR2300077379).

## Conflicts of Interest

The authors declare no conflicts of interest.

## Supporting information


**Table S1:** The list of institutions and ethics batch number.
**Table S2:** Details of immune‐related adverse and management in patients with autoimmune diseases.
**Table S3:** Incidence of immune‐related adverse according to category and grade.
**Table S4:** Incidence and grades, management, category of irAEs across biological subtypes.
**Table S5:** Management and outcomes of immune‐related adverse.
**Table S6:** Risk factors for irAEs and severe irAEs with univariable and multivariable logistic regression analysis.
**Table S7:** Immune‐related adverse according to different anti‐PD‐1 based therapies.
**Table S8:** The impact of different anti‐PD‐1 based combination therapy on any grade irAEs, stratified by sex, age, biologic subtypes, number of metastases, anti‐PD‐1.
**Table S9:** The impact of different anti‐PD‐1 based combination therapy on grade 3–5 irAEs, stratified by sex, age, biologic subtypes, number of metastases, anti‐PD‐1.

## Data Availability

Research data are not shared.
