# Supplementary material for: Risk Factors and Comparative Safety of Anti‐PD‐1 Combination Therapies in Advanced Melanoma: A Nationwide Real‐World Cohort Study From China
Source: Cancer Med. 2026 Apr 14;15(4):e71694. doi: 10.1002/cam4.71694 (PMC13079074; doi:10.1002/cam4.71694)
Supplement: Supplementary file 1 — Table S1: The list of institutions and ethics batch number. Table S2: Details of immune‐related adverse and management in patients with autoimmune diseases. Table S3: Incidence of immune‐related adverse according to category and grade. Table S4: Incidence and grades, management, category of irAEs across biological subtypes. Table S5: Management and outcomes of immune‐related adverse. Table S6: Risk factors for irAEs and severe irAEs with univariable and multivariable logistic regression analysis. Table S7: Immune‐related adverse according to different anti‐PD‐1 based therapies. Table S8: The impact of different anti‐PD‐1 based combination therapy on any grade irAEs, stratified by sex, age, biologic subtypes, number of metastases, anti‐PD‐1. Table S9: The impact of different anti‐PD‐1 based combination therapy on grade 3–5 irAEs, stratified by sex, age, biologic subtypes, number of metastases, anti‐PD‐1. [file CAM4-15-e71694-s001.docx]

Table S1.The list of institutions and ethics batch number

|  | Institution/ hospital | Name of the ethic committee | Ethics batch number |
| --- | --- | --- | --- |
| Primary sponsor | Medical Department, Xi'an Jiaotong University | Biomedical Ethics Committee of Medical Department, Xi'an Jiaotong University | No.2023-2093 |
| Secondary sponsor | Center for Drug Safety and Policy Research, Xi’an Jiaotong University |  |  |
| 1 | The First Affiliated Hospital of Xi'an Jiaotong University | Ethics Committee of The First Affiliated Hospital of Xi’an Jiao University | No.XJTU1AF2024LSK-002 |
| 2 | Xijing Hospital, Fourth Military Medical University | The medical Ethics Committee of the First Affiliated Hospital of the Air Force Medical University | NO.KY20232399-C-1 |
| 3 | Gansu Provincial Cancer Hospital | Ethics Committee of Gansu Provincial Cancer Hospital | No.P202312080089 |
| 4 | The affiliated Cancer Hospital of Xinjiang Medical University | Ethics Committee of the Affiliated Cancer Hospital of Xinjiang Medical University | No. XJZ-CR-2023-003 |
| 5 | Henan Cancer Hospital | Medical Ethics Committee of Henan Cancer Hospital | No.2023-519-001 |
| 6 | Shandong Cancer Hospital and Institute | Ethics Committee of Affiliated Cancer Hospital of Shandong First Medical University | SDTHEC2023-012-004 |
| 7 | Nanjing Drum Tower Hospital | Medical Ethics Committee of Nanjing Drum Tower Hospital | No.2024-153-02 |

Table S2. Details of immune-related adverse and management in patients with autoimmune diseases

| **Patient** | Autoimmune diseases | Whether receiving treatment for autoimmune disease (and duration of therapy) | irAEs | Number of irAEs | | | | | Duration  of PD-1（month） | Management | | |
| --- | --- | --- | --- | --- | --- | --- | --- | --- | --- | --- | --- | --- |
|  |  |  |  | Total | Grade1 | Grade2 | Grade3 | Grade4 |  | Steroid | Stop  treatment | Hospital |
| 1 | Ankylosing spondylitis | No | thyroid dysfunction | 1 | 1 |  |  |  | 3.5 |  |  |  |
| 2 | Ankylosing spondylitis | No | none | 0 |  |  |  |  | 2.0 |  |  |  |
| 3 | Thyroiditis;  Adrenocortical hypofunction | Yes, Levothyroxine for 4 years, prednisone for 6 months | erythema multiforme; Vitiligo; hypertriglyceridemia; hyperglycemia; cholesterol high | 5 | 4 |  | 1 |  | 21.6 | Yes |  | Yes |
| 4 | Adrenocortical hypofunction; Vitiligo | Yes, Prednisone acetate 5mg qd for 2 years | vomiting; hypertriglyceridemia; gamma glutamyl transferase | 3 | 2 |  | 1 |  | 6.5 | Yes |  |  |
| 5 | Adrenocortical hypofunction; | Yes, Prednisone acetate 5mg qd for 1.5 years | atrial fibrillation; thyroid dysfunction; hypertriglyceridemia; cholesterol high | 4 | 2 | 2 |  |  | 5.3 | Yes | Yes |  |
| 6 | Thyroiditis;  Adrenocortical hypofunction | No | Thrombocytopenia; hyperglycemia; hypertriglyceridemia; cholesterol high | 4 | 1 | 2 | 1 |  | 1.9 | Yes | Yes | Yes |
| 7 | Vitiligo | No | Maculopapule ; Pustular rash; cheilitis; elevated cardiac troponin I | 4 | 2 | 2 |  |  | 19.6 | Yes |  |  |
| 8 | Thyroiditis；Vitiligo | No | erythema; rash; cutaneous infection; thyroid dysfunction; | 4 |  | 3 | 1 |  | 24.6 | Yes |  | Yes |
| 9 | psoriasis | No | arthralgia; hypertriglyceridemia | 2 | 1 | 1 |  |  | 12.8 |  |  |  |
| 10 | psoriasis; lichen planus; adrenal cortical insufficiency | Yes, tacrolimus ointment for 1 years | bullous pemphigoid; Recurrence of lichen planus | 2 |  |  | 2 |  | 8.5 | Yes | Yes | Yes |
| 11 | lichen planus | No | adrenocortical hypofunction; thyroid dysfunction; hypertriglyceridemia; hyperglycemia;  abnormal liver function | 5 | 3 | 2 |  |  | 6.5 | Yes |  |  |
| 12 | Vitiligo; asthma | No | vertigo; elevated troponin levels; hypertriglyceridemia; cholesterol high | 4 | 3 | 1 |  |  | 10.2 |  |  |  |
| 13 | Vitiligo | No | Pruritus;hypertriglyceridemia | 1 | 1 |  |  |  | 2.2 |  |  |  |
| 14 | lichen planus | No | pneumonia; recurrence of lichen planus; Vitiligo | 3 | 1 | 2 |  |  | 7.4 | Yes | Yes |  |
| 15 | Vitiligo | No | abnormal liver function; hypertriglyceridemia; | 3 | 2 |  | 1 |  | 5.1 |  |  |  |
| 16 | Psoriasis | No | Psoriasis recurrence; thyroid dysfunction; | 2 |  | 2 |  |  | 28.9 | Yes | 0 | 0 |

Table S3. Incidence of immune-related adverse according to category and grade.

|  | Any grade, n (%) | Grade 1-2, n (%) | Grade 3-4, n (%) |
| --- | --- | --- | --- |
| Total number of patients with irAEs | 336 (66.1%) | 322 (63.4%) | 70 (13.8%) |
| **irAEs by organ** |  |  |  |
| **Endocrine** | 207 (40.7) | 205 (40.4) | 3 (0.6) |
| thyroid dysfunction | 204 (40.2) | 203 (40.0) | 1 (0.2) |
| adrenal insufficiency | 9 (1.8) | 7 (1.4) | 2 (0.4) |
| testosterone deficiency | 1 (0.2) | 1 (0.2) | 0 (0.0) |
| **Metabolism** | 113 (22.2) | 106 (20.9) | 7 (1.4) |
| hyperglycaemia | 32 (6.3) | 31 (6.1) | 1 (0.2) |
| hypertriglyceridemia | 72 (14.2) | 69 (13.6) | 3 (0.6) |
| hyperuricemia | 29 (5.7) | 29 (5.7) | 0 (0.0) |
| other | 6 (1.2) | 3 (0.6) | 3 (0.6) |
| **Investigations** | 96 (18.9) | 89 (17.5) | 8 (1.6) |
| cholesterol high | 34 (6.7) | 34 (6.7) | 0 (0.0) |
| creatine phosphokinase increased | 35 (6.9) | 34 (6.7) | 1 (0.2) |
| gamma glutamyl transferase | 28 (5.5) | 22 (4.3) | 6 (1.2) |
| lipase increased, other | 8 (1.6) | 7 (1.4) | 1 (0.2) |
| **Skin** | 79 (15.6) | 75 (14.8) | 5 (1.0) |
| vitiligo | 25 (4.9) | 25 (4.9) | 0 (0.0) |
| pruritus | 15 (3.0) | 14 (2.8) | 1 (0.2) |
| rash | 26 (5.1) | 25 (4.9) | 1 (0.2) |
| psoriasis, erythema,other | 25 (4.9) | 21 (4.1) | 4 (0.8) |
| **Hepatobiliary** | 36 (7.1) | 31 (6.1) | 6 (1.2) |
| **Gastrointestinal** | 36 (7.1) | 31 (6.1) | 7 (1.4) |
| **Hematologic** | 29 (5.7) | 21 (4.1) | 10 (2.0) |
| thrombocytopenia | 17 (3.3) | 10 (2.0) | 7 (1.4) |
| white blood cell count | 12 (2.4) | 7 (1.4) | 5 (1.0) |
| hemoglobin, other | 7 (1.4) | 6 (1.2) | 2 (0.4) |
| **Renal and urinary** | 28 (5.5) | 23 (4.5) | 6 (1.2) |
| **General** | 18 (3.5) | 18 (3.5) | 0 (0.0) |
| fever | 11 (2.2) | 11 (2.2) | 0 (0.0) |
| fatigue | 7 (1.4) | 7 (1.4) | 0 (0.0) |
| other | 1 (0.2) | 1 (0.2) | 0 (0.0) |
| **Respiratory** | 16 (3.1) | 6 (1.2) | 10 (2.0) |
| pneumonitis | 13 (2.6) | 3 (0.6) | 10 (2.0) |
| cough, other | 3 (0.6) | 3 (0.6) | 0 (0.0) |
| **Vascular** | 14 (2.8) | 12 (2.4) | 2 (0.4) |
| **Infections** | 8 (1.6) | 1 (0.2) | 7 (1.4) |
| **Musculoskeletal** | 9 (1.8) | 8 (1.6) | 1 (0.2) |
| **Cardiac disorders** | 6 (1.2) | 3 (0.6) | 3 (0.6) |
| **Nervous system** | 5 (1.0) | 4 (0.8) | 1 (0.2) |
| **Ear and labyrinth** | 1 (0.2) | 1 (0.2) | 0 (0.0) |
| **Eye** | 1 (0.2) | 1 (0.2) | 0 (0.0) |
| **Reproductive system and breast** | 1 (0.2) | 1 (0.2) | 0 (0.0) |

**Table S4.** Incidence and grades, management, category of irAEs across biological subtypes

|  | Number of patients (%) | | | | | | P |
| --- | --- | --- | --- | --- | --- | --- | --- |
|  | Total | Cutaneous | Acral | Mucosal | Uvea | Unknow |  |
|  | 508 | 111 (22) | 194 (38) | 158 (31) | 11 (2) | 34 (7) |  |
| **irAEs** |  |  |  |  |  |  | 0.655 |
| No | 172(34) | 33 (30) | 72 (37) | 51 (32) | 3 (27) | 13 (38) |  |
| Yes | 336(66) | 78 (70) | 122 (63) | 107 (68) | 8 (73) | 21 (62) |  |
| **Grades of irAEs** |  |  |  |  |  |  | 0.384 |
| Without irAE | 172(34) | 33 (30) | 72 (37) | 51 (32) | 3 (27) | 13 (38) |  |
| Grade 1-2 | 266(52) | 62 (56) | 104 (54) | 79 (50) | 5 (46) | 16 (47) |  |
| Grade 3-4 | 70 (14) | 16 (14) | 18 (9) | 28 (18) | 3 (27) | 5 (15) |  |
| **Management of irAEs** |  |  |  |  |  |  |  |
| Steroid | 38 (7) | 10 (9) | 12 (6) | 12 (8) | 1 (9) | 3 (9) | 0.913 |
| Discontinuation of PD-1 | 40 (8) | 9 (8) | 9 (5) | 16 (10) | 2 (18) | 4 (12) | 0.182 |
| Hospital | 21 (4) | 5 (5) | 10 (5) | 3 (2) | 1 (9) | 2 (6) | 0.480 |
| **Category of irAEs by organ** |  |  |  |  |  |  |  |
| **Endocrine** |  |  |  |  |  |  |  |
| thyroid dysfunction | 204 (40) | 51 (46) | 67 (35) | 66 (42) | 7 (64) | 13 (38) | 0.144 |
| **Skin** | 79 (16) | 17 (15) | 38 (20) | 18 (11) | 2 (18) | 4 (12) | 0.295 |
| **Laboatory abnormalities** |  |  |  |  |  |  |  |
| Hypertriglyceridemia | 72 (16) | 20 (18) | 28 (14) | 19 (12) | 1 (9) | 4 (12) | 0.671 |
| Creatine phosphokinase increased | 36 (7) | 8 (7) | 14 (7) | 12 (8) | 1 (9) | 1 (3) | 0.906 |
| Cholesterol high | 35 (7) | 10 (9) | 8 (4) | 12 (8) | 0 (0) | 5 (15) | 0.123 |
| **Hepatobiliary** | 61 (12) | 15 (14) | 18 (9) | 20 (13) | 3 (27) | 5 (15) | 0.363 |
| **Gastrointestinal** | 36 (7) | 10 (9) | 8 (4) | 15 (9) | 2 (18) | 1 (3) | 0.110 |
| **Hematologic** | 29 (6) | 7 (6) | 10 (5) | 10 (6) | 0 (0) | 2 (6) | 0.915 |
| **Renal and urinary** | 28 (6) | 5 (5) | 7 (4) | 15 (9) | 1 (9) | 0 (0) | 0.071 |
| **Pneumonitis** | 13 (3) | 3 (3) | 2 (1) | 4 (3) | 1 (9) | 3 (9) | 0.060 |

Table S5. Management and outcomes of immune-related adverse.

|  | No. of irAEs, n (%) | Steroid, n (%) | Discontinuation of PD-1, n (%) | Hospital, n (%) |
| --- | --- | --- | --- | --- |
| **Total** | 775 | 50 (6.5) | 45 (5.8) | 23 (3.0) |
| Grade 1-2 | 696 (89.8) | 28 (4.0) | 12 (1.7) | 1 (0.1) |
| Grade 3-4 | 79 (10.2) | 22 (27.8) | 33 (41.8) | 22 (27.9) |
| **Endocrine** | 210 | 10 (4.8) | 5 (2.4) | 1 (0.5) |
| thyroid dysfunction | 198 | 2 (1.0) | 3 (1.5) | 0 (0.0) |
| Grade 1-2 | 197 (99.5) | 1 (0.5) | 2 (1.0) | 0 (0.0) |
| Grade 3-4 | 1 (0.5) | 1 (100) | 1 (100) | 0 (0.0) |
| adrenal insufficiency | 10 | 8 (80) | 2 (20) | 1 (10) |
| Grade 1-2 | 8 (80.0) | 6 (75) | 0 | 0 (0.0) |
| Grade 3-4 | 2 (20.0) | 2 (100) | 2 (100) | 1 (50) |
| **Skin** | 93 | 21 (22.6) | 6 (6.5) | 5 (5.4) |
| Grade 1-2 | 87 (93.5) | 15 (17.2) | 4 (4.6) | 0 (0.0) |
| Grade 3-4 | 6 (6.5) | 6 (100) | 2 (33.3) | 5 (83.3) |
| **Metabolism** | 143 | 0 (0.0) | 2 (1.4) | 0 (0.0) |
| hyperglycaemia | 36 | 0 (0.0) | 1 (2.8) | 0 (0.0) |
| Grade 3-4 | 1 (2.8) | 0 (0.0) | 1 (100) | 0 (0.0) |
| hypertriglyceridemia | 72 | 0 (0.0) | 1 (1.4) | 0 (0.0) |
| Grade 3-4 | 3 (4.2) | 0 (0.0) | 1 (33.3) | 0 (0.0) |
| **Hepatobiliary** | 39 | 2 (5.1) | 3 (7.7) | 0 (0.0) |
| Grade 1-2 | 32 (82.1) | 1 (3.1) | 2 (6.3) | 0 (0.0) |
| Grade 3-4 | 7 (17.9) | 1 (14.3) | 1 (14.3) | 0 (0.0) |
| **Gastrointestinal** | 36 | 2 (5.6) | 2 (5.6) | 1 (2.8) |
| Grade 1-2 | 30 (83.3) | 1 (3.3) | 0 (0.0) | 0 (0.0) |
| Grade 3-4 | 6 (16.7) | 1 (16.7) | 2 (33.3) | 1 (16.7) |
| **Hematologic** | 36 | 4 (11.1) | 11 (30.6) | 5 (13.9) |
| thrombocytopenia | 17 | 2 (11.8) | 7 (41.2) | 3 (17.6) |
| Grade 3-4 | 7 (41.2) | 2 (28.6) | 7 (100) | 3 (42.9) |
| white blood cell count | 11 | 1 (9.1) | 2 (18.2) | 1 (9.1) |
| Grade 3-4 | 5 (45.5) | 1 (20) | 2 (40) | 1 (20) |
| hemoglobin, other | 8 | 1 (12.5) | 2 (25) | 1 (12.5) |
| Grade 3-4 | 2 (25) | 1 (50) | 2 (100) | 1 (50) |
| **Renal and urinary** | 29 | 1 (3.5) | 1 (3.5) | 0 (0.0) |
| Grade 3-4 | 6 (20.7) | 1 (16.7) | 1 (16.7) | 0 (0.0) |
| **General** | 20 | 1 (5.0) | 1 (5.0) | 0 (0.0) |
| fever | 11 | 1 (9.1) | 0 (0.0) | 0 (0.0) |
| Grade 1-2 | 11 (100) | 1 (9.1) | 0 (0.0) | 0 (0.0) |
| **Respiratory** | 17 | 3 (17.6) | 8 (47.1) | 6 (35.3) |
| pneumonitis | 12 | 3 (25) | 8 (66.7) | 6 (50) |
| Grade 1-2 | 3 (25) | 0 (0.0) | 1 (33.3) | 1 (33.3) |
| Grade 3-4 | 9 (75) | 3 (33.3) | 7 (77.8) | 5 (55.6) |
| **Infections** | 9 | 1 (11.1) | 1 (11.1) | 4 (44.4) |
| Grade 3-4 | 7 (77.8) | 1 (14.3) | 1 (14.3) | 4 (57.1) |
| **Musculoskeletal** | 9 | 1 (11.11) | 0 (0.0) | 0 (0.0) |
| Grade 3-4 | 1 (11.1) | 1 (100) | 0 (0.0) | 0 (0.0) |
| **Cardiac** | 6 | 2 (33.3) | 5 (83.3) | 1 (16.7) |
| Grade 1-2 | 3 (50) | 1 (33.3) | 2 (66.7) | 0 (0.0) |
| Grade 3-4 | 3 (50) | 1 (33.3) | 3 (100) | 1(33.3) |

Table S6. Risk factors for irAEs and severe irAEs with univariable and multivariable logistic regression analysis.

|  |  | Univariable analysis | | | | | |  | Multivariable analysis | | | | | |
| --- | --- | --- | --- | --- | --- | --- | --- | --- | --- | --- | --- | --- | --- | --- |
|  |  | Any grade | | Grade 1-2 | | Grade 3-5 | |  | Any grade | | Grade 1-2 | | Grade 3-5 | |
|  | n | OR (95% Cl) | P | OR (95% Cl) | P | OR (95% Cl) | P |  | OR (95% Cl) | P | OR (95% Cl) | P | OR (95% Cl) | P |
| **Sex** Male | 252 | Reference |  | Reference |  | Reference |  |  | Reference |  | Reference |  | Reference |  |
| Female | 256 | 1.2 (0.8, 1.8) | 0.287 | 1.3 (0.9, 1.9) | 0.155 | 1.0 (0.6, 1.7) | 0.852 |  | 1.1 (0.7, 1.7) | 0.772 | 1.1 (0.7, 1.7) | 0.710 | 1.0 (0.5, 2.0) | 0.927 |
| **Age** ≤65 | 340 | Reference |  | Reference |  | Reference |  |  | Reference |  | Reference |  | Reference |  |
| >65 | 168 | 1.2 (0.8, 1.7) | 0.439 | 1.2 (0.8, 1.8) | 0.281 | 0.6 (0.3, 1.1) | 0.095 |  | 1.6 (1.0, 2.6) | 0.034 | 1.7 (1.1, 2.8) | 0.017 | 0.7 (0.4, 1.4) | 0.358 |
| **ECOG PS** 0-1 | 473 | Reference |  | Reference |  | Reference |  |  | Reference |  | Reference |  | Reference |  |
| ≥2 | 35 | 0.3 (0.1, 0.7) | 0.007 | 0.3 (0.1, 0.6) | 0.002 | 1.6 (0.6, 4.4) | 0.359 |  | 0.6 (0.2, 1.4) | 0.230 | 0.5 (0.2, 1.2) | 0.109 | 2.6 (0.8, 8.3) | 0.101 |
| **Stage**  Ⅲ | 63 | Reference |  | Reference |  | Reference |  |  | Reference |  | Reference |  | Reference |  |
| Ⅳ | 445 | 1.3 (0.8, 2.3) | 0.298 | 1.2 (0.7, 2.0) | 0.589 | 2.6 (0.9, 7.3) | 0.077 |  | 1.5 (0.8, 3.0) | 0.214 | 1.4 (0.7, 2.7) | 0.337 | 2.8 (0.9, 8.8) | 0.074 |
| **Subtypes** Cutaneous | 111 | Reference |  | Reference |  | Reference |  |  | Reference |  | Reference |  | Reference |  |
| Acral | 194 | 0.7 (0.4, 1.1) | 0.143 | 0.7 (0.4, 1.2) | 0.207 | 0.6 (0.3, 1.2) | 0.173 |  | 0.7 (0.4, 1.3) | 0.322 | 0.8 (0.5, 1.5) | 0.516 | 0.6 (0.2, 1.4) | 0.269 |
| Mucosal | 158 | 0.8 (0.5, 1.4) | 0.477 | 0.9 (0.5, 1.4) | 0.5364 | 1.3 (0.7, 2.5) | 0.471 |  | 0.8 (0.4, 1.5) | 0.528 | 0.8 (0.5, 1.6) | 0.600 | 1.7 (0.8, 3.7) | 0.169 |
| Uvea | 11 | 1.1 (0.3, 4.3) | 0.913 | 1.3 (0.3, 5.1) | 0.7269 | 2.2 (0.5, 9.3) | 0.272 |  | 0.9 (0.2, 4.5) | 0.898 | 1.1 (0.2, 5.6) | 0.899 | 2.7 (0.6, 12.7) | 0.213 |
| Unkown | 34 | 0.7 (0.3, 1.5) | 0.301 | 0.8 (0.3, 1.7) | 0.532 | 1.0 (0.3, 3.0) | 0.966 |  | 1.0 (0.4, 2.4) | 0.938 | 1.2 (0.5, 3.1) | 0.665 | 1.5 (0.5, 4.8) | 0.504 |
| **No. of metastases** <3 | 358 | Reference |  | Reference |  | Reference |  |  | Reference |  | Reference |  | Reference |  |
| ≥3 | 150 | 0.6 (0.4, 0.9) | 0.022 | 0.6 (0.4, 0.9) | 0.015 | 1.0 (0.6, 1.8) | 0.926 |  | 0.8 (0.5, 1.3) | 0.298 | 0.8 (0.5, 1.4) | 0.484 | 0.9 (0.5, 1.8) | 0.835 |
| **Brain metastases**  No | 451 | Reference |  | Reference |  | Reference |  |  | Reference |  | Reference |  | Reference |  |
| Yes | 57 | 0.6 (0.3, 1.0) | 0.049 | 0.4 (0.3, 0.8) | 0.004 | 1.4 (0.7, 2.9) | 0.383 |  | 0.8 (0.4, 1.5) | 0.469 | 0.6 (0.3, 1.2) | 0.157 | 1.5 (0.7, 3.6) | 0.327 |
| **Liver metastasis** No | 388 | Reference |  | Reference |  | Reference |  |  | Reference |  | Reference |  | Reference |  |
| Yes | 120 | 0.8 (0.5, 1.2) | 0.237 | 0.8 (0.5, 1.1) | 0.189 | 1.0 (0.6, 1.9) | 0.888 |  | 0.8 (0.5, 1.4) | 0.465 | 0.8 (0.5, 1.4) | 0.486 | 0.7 (0.4, 1.5) | 0.363 |
| **Cardiovascular disease** No | 333 | Reference |  | Reference |  | Reference |  |  | Reference |  | Reference |  | Reference |  |
| Yes | 175 | 1.4 (0.9, 2.1) | 0.104 | 1.3 (0.9, 1.9) | 0.171 | 1.0 (0.6, 1.7) | 0.975 |  | 1.5 (0.9, 2.5) | 0.093 | 1.4 (0.9, 2.3) | 0.159 | 1.2 (0.6, 2.2) | 0.641 |
| **Lung disease** No | 483 | Reference |  | Reference |  | Reference |  |  | Reference |  | Reference |  | Reference |  |
| Yes | 25 | 2.1 (0.8, 5.7) | 0.141 | 1.5 (0.6, 3.7) | 0.362 | 2.1 (0.8, 5.4) | 0.136 |  | 3.1 (1.0, 9.8) | 0.0591 | 2.1 (0.7, 6.2) | 0.165 | 2.9 (1.0, 8.4) | 0.052 |
| **Liver/kidneydysfunction** No | 458 | Reference |  | Reference |  | Reference |  |  | Reference |  | Reference |  | Reference |  |
| Yes | 50 | 0.9 (0.5, 1.7) | 0.736 | 0.6 (0.4, 1.2) | 0.1491 | 2.5 (1.2, 4.9) | 0.010 |  | 1.4 (0.7, 2.8) | 0.348 | 1.0 (0.5, 2.0) | 0.973 | 2.9 (1.3, 6.5) | 0.009 |
| **Autoimmune disease** No | 492 | Reference |  | Reference |  | Reference |  |  | Reference |  | Reference |  | Reference |  |
| Yes | 16 | 3.7 (0.8, 16.5) | 0.086 | 2.6 (0.7, 9.1) | 0.145 | 4.0 (1.4, 11.4) | 0.009 |  | 2.1 (0.4, 10.1) | 0.377 | 1.4 (0.3, 5.6) | 0.649 | 2.7 (0.8, 8.7) | 0.100 |
| **Prior ICIs** No | 370 | Reference |  | Reference |  | Reference |  |  | Reference |  | Reference |  | Reference |  |
| Yes | 138 | 0.9 (0.6, 1.3) | 0.490 | 0.8 (0.5, 1.2) | 0.258 | 1.7 (1.0, 2.9) | 0.045 |  | 0.7 (0.4, 1.2) | 0.185 | 0.7 (0.4, 1.1) | 0.140 | 1.4 (0.7, 2.6) | 0.331 |
| **Prior** **cytokine** No | 334 | Reference |  | Reference |  | Reference |  |  | Reference |  | Reference |  | Reference |  |
| Yes | 174 | 1.5 (1.0, 2.3) | 0.032 | 1.6 (1.1, 2.3) | 0.024 | 1.4 (0.9, 2.4) | 0.175 |  | 1.2 (0.7, 2.0) | 0.423 | 1.3 (0.8, 2.2) | 0.288 | 1.1 (0.6, 2.0) | 0.864 |
| **Prior TKI**  No | 445 | Reference |  | Reference |  | Reference |  |  | Reference |  | Reference |  | Reference |  |
| Yes | 63 | 1.9 (1.0, 3.6) | 0.040 | 1.5 (0.8, 2.7) | 0.159 | 2.5 (1.3, 4.6) | 0.005 |  | 1.4 (0.7, 3.1) | 0.382 | 1.2 (0.6, 2.5) | 0.688 | 2.4 (1.1, 5.1) | 0.024 |
| **Prior anti-VEGF** No | 470 | Reference |  | Reference |  | Reference |  |  | Reference |  | Reference |  | Reference |  |
| Yes | 38 | 1.0 (0.5, 2.0) | 0.962 | 1.0 (0.5, 2.0) | 0.976 | 1.2 (0.5, 3.0) | 0.709 |  | 0.9 (0.4, 2.1) | 0.859 | 1.2 (0.5, 2.7) | 0.734 | 0.7 (0.3, 2.1) | 0.541 |
| **Line of therapy** 1 line | 347 | Reference |  | Reference |  | Reference |  |  | Reference |  | Reference |  | Reference |  |
| 2 line | 125 | 1.5 (1.0, 2.4) | 0.075 | 1.4 (0.9, 2.2) | 0.128 | 1.6 (0.9, 2.8) | 0.105 |  | 1.6 (1.0, 2.7) | 0.063 | 1.5 (0.9, 2.6) | 0.092 | 1.7 (0.9, 3.2) | 0.092 |
| ≥3 line | 36 | 1.0 (0.5, 2.0) | 0.992 | 0.8 (0.4, 1.5) | 0.454 | 1.8 (0.7, 4.4) | 0.194 |  | 1.2 (0.5, 2.7) | 0.674 | 1.0 (0.4, 2.2) | 0.928 | 1.5 (0.6, 3.9) | 0.421 |
| **Anti-PD-1** Toripalimab | 425 | Reference |  | Reference |  | Reference |  |  | Reference |  | Reference |  | Reference |  |
| Pembrolizumab | 83 | 1.4 (0.8, 2.4) | 0.197 | 1.3 (0.8, 2.2) | 0.275 | 2.4 (1.3, 4.3) | 0.004 |  | 1.2 (0.6, 2.1) | 0.601 | 1.0 (0.5, 1.8) | 0.966 | 2.6 (1.4, 5.1) | 0.004 |
| **Chemotherapy**  No | 353 | Reference |  | Reference |  | Reference |  |  | Reference |  | Reference |  | Reference |  |
| Yes | 155 | 0.8 (0.4, 1.1) | 0.061 | 0.6 (0.3, 1.0) | 0.043 | 0.9 (0.5, 1.6) | 0.704 |  | 0.9 (0.5, 1.4) | 0.569 | 0.7 (0.4, 1.3) | 0.138 | 1.8 (0.9, 3.8) | 0.104 |
| **DurationofPD-1** 0-24 month | 465 | Reference |  | Reference |  | Reference |  |  | Reference |  | Reference |  | Reference |  |
| >24 months | 43 | 2.8 (1.2, 6.5) | 0.014 | 3.2 (1.4, 7.4) | 0.006 | 1.2 (0.5, 2.9) | 0.620 |  | 2.6 (1.1, 6.3) | 0.036 | 3.2 (1.3, 7.8) | 0.011 | 1.0 (0.4, 2.5) | 0.981 |
| **Combination therapy** |  |  |  |  |  |  |  |  |  |  |  |  |  |  |
| PD-1 monotherapy | 181 | Reference |  | Reference |  | Reference |  |  | Reference |  | Reference |  | Reference |  |
| PD-1+TKI | 131 | 1.9 (1.2, 3.0) | 0.008 | 1.8 (1.2, 3.0) | 0.008 | 1.5 (0.8, 3.1) | 0.232 |  | 2.1 (1.2, 3.6) | 0.006 | 1.9 (1.1, 3.3) | 0.015 | 1.5 (0.7, 3.5) | 0.313 |
| PD-1+anti-VEGF | 56 | 1.7 (0.9, 3.1) | 0.096 | 1.7 (0.9, 3.1) | 0.096 | 1.2 (0.4, 3.1) | 0.770 |  | 2.5 (1.2, 5.0) | 0.012 | 2.6 (1.3, 5.2) | 0.007 | 0.7 (0.2, 2.3) | 0.596 |
| PD-1+ IFN-α1b | 99 | 3.8 (2.1, 6.6) | <0.001 | 3.2 (1.4, 7.4) | 0.006 | 2.1 (1.0, 4.4) | 0.036 |  | 3.6 (1.8, 6.9) | <0.001 | 3.1 (1.6, 5.9) | <0.001 | 2.2 (0.9, 5.3) | 0.067 |
| PD-1+IFN-α1b +anti-VEGF | 41 | 4.9 (2.1, 11.7) | <0.001 | 3.2 (1.4, 7.4) | 0.006 | 3.5 (1.5, 8.3) | 0.004 |  | 4.3 (1.7, 11.1) | 0.003 | 4.4 (1.7, 11.5) | 0.002 | 3.3 (1.1, 9.7) | 0.028 |

Multivariable regression adjusted age, gender, ECOG PS, stage, biologic subtypes, number of metastases, brain metastases, Liver/kidney dysfunction, autoimmune disease, prior ICIs, prior cytokine, prior TKI, line of therapy, whether chemotherapy was added, anti-PD-1, duration of anti-PD-1, different combination therapies

Table S7. Immune-related adverse according to different anti-PD-1 based therapies.

|  | PD-1 monotherapy（n=181), No.(%) | | | PD-1 combined with TKI（n=131), No.(%) | | | PD-1 combined with anti-VEGF（n=56), No.(%) | | | PD-1 combined with IFN-α1b（n=99), No.(%) | | | PD-1 combined with TKI and IFN-α1b（n=41), No.(%) | | | P1 | P2 | P3 | |  |
| --- | --- | --- | --- | --- | --- | --- | --- | --- | --- | --- | --- | --- | --- | --- | --- | --- | --- | --- | --- | --- |
|  | Any grade | Grade 1-2 | Grade 3-4 | Any grade | Grade 1-2 | Grade 3-4 | Any grade | Grade 1-2 | Grade 3-4 | Any grade | Grade 1-2 | Grade 3-4 | Any grade | Grade 1-2 | Grade 3-4 |  |  |  |  |  |
| Total number of patients with irAE | 94（51.9） | 90 (49.7) | 17 (9.4) | 89 (67.9) | 85 (64.9) | 18 (13.7) | 38 (67.9) | 35 (62.5) | 6 (10.7) | 81 (81.8) | 78 (78.8) | 18 (18.2) | 34 (82.9) | 34 (82.9) | 11 (26.8) | <0.001 | <0.001 | 0.028 | |  |
| **Endocrine** | 55 (30.4) | 55 (30.4) | 0 (0.0) | 61 (46.6) | 60 (45.8) | 1 (0.8) | 18 (32.1) | 18 (32.1) | 0 (0.0) | 47 (47.5) | 46 (46.5) | 2 (2.0) | 26 (63.4) | 26 (63.4) | 0 (0.0) | <0.001 | <0.001 | 0.271 | |  |
| hypothyroidism/ hyperthyroidism | 55 (30.4) | 55 (30.4) | 0 (0.0) | 61 (46.6) | 60 (45.8) | 1 (0.8) | 17 (30.4) | 17 (30.4) | 0 (0.0) | 46 (46.5) | 46 (46.5) | 0 (0.0) | 25 (61.0) | 25 (61.0) | 0 (0.0) | <0.001 | <0.001 | 0.578 | |  |
| adrenal insufficiency | 1 (0.6) | 1 (0.6) | 0 (0.0) | 0 (0.0) | 0 (0.0) | 0 (0.0) | 1 (1.8) | 1 (1.8) | 0 (0.0) | 6 (6.1) | 4 (4.0) | 2 (2.0) | 1 (2.4) | 1 (2.4) | 0 (0.0) | 0.006 | 0.081 | 0.081 | |  |
| testosterone deficiency | 0 (0.0) | 0 (0.0) | 0 (0.0) | 1 (0.8) | 1 (0.8) | 0( 0.0) | 0 (0.0) | 0 (0.0) | 0 (0.0) | 0 (0.0) | 0 (0.0) | 0 (0.0) | 0 (0.0) | 0 (0.0) | 0 (0.0) | 0.578 | 0.578 |  | |  |
| **Metabolism** | 21 (11.6) | 21 (11.6) | 0 (0.0) | 20 (15.3) | 18 (13.7) | 2 (1.5) | 14 (25.0) | 14 (25.0) | 0 (0.0) | 36 (36.4) | 34 (34.3) | 2 (2.0) | 22 (53.7) | 19 (46.3) | 3 (7.3) | <0.001 | <0.001 | 0.006 | |  |
| hyperglycaemia | 9 (5.0) | 9 (5.0) | 0 (0.0) | 3 (2.3) | 3 (2.3) | 0 (0.0) | 2 (3.6) | 2 (3.6) | 0 (0.0) | 12 (12.1) | 11 (11.1) | 1 (1.0) | 6 (14.6) | 6 (14.6) | 0 (0.0) | 0.004 | 0.008 | 0.387 |  |  |
| hypertriglyceridemia | 8 (4.4) | 8 (4.4) | 0 (0.0) | 10 (7.6) | 9 (6.9) | 1 (0.8) | 6 (10.7) | 6 (10.7) | 0 (0.0) | 28 (28.3) | 28 (28.3) | 0 (0.0) | 20 (48.8) | 18 (43.9) | 2 (4.9) | <0.001 | <0.001 | 0.005 |  |  |
| hyperuricemia | 4 (2.2) | 4 (2.2) | 0 (0.0) | 7 (5.3) | 7 (5.3) | 0 (0.0) | 7 (12.5) | 7 (12.5) | 0 (0.0) | 8 (8.1) | 8 (8.1) | 0 (0.0) | 3 (7.3) | 3 (7.3) | 0 (0.0) | 0.038 | 0.038 |  |  |  |
| other | 2 (1.1) | 2 (1.1) | 0 (0.0) | 2 (1.5) | 1 (0.8) | 1 (0.8) | 0 (0.0) | 0 (0.0) | 0 (0.0) | 1 (1.0) | 0 (0.0) | 1 (1.0) | 1 (2.4) | 0 (0.0) | 1 (2.4) | 0.845 | 0.727 | 0.385 |  |  |
| **Investigations** | 28 (15.5) | 26 (14.4) | 3 (1.7) | 32 (24.4) | 28 (21.4) | 4 (3.1) | 12 (21.4) | 11 (19.6) | 1 (1.8) | 17 (17.2) | 17 (17.2) | 0 (0.0) | 7 (17.1) | 7 (17.1) | 0 (0.0) | 0.341 | 0.595 | 0.391 |  |  |
| cholesterol high | 5 (2.8) | 5 (2.8) | 0 (0.0) | 10 (7.6) | 10 (7.6) | 0 (0.0) | 5 (8.9) | 5 (8.9) | 0 (0.0) | 9 (9.1) | 9 (9.1) | 0 (0.0) | 5 (12.2) | 5 (12.2) | 0 (0.0) | 0.091 | 0.091 |  |  |  |
| Creatinephosphokinase increased | 12 (6.6) | 12 (6.6) | 0 (0.0) | 16 (12.2) | 15 (11.5) | 1 (0.8) | 2 (3.6) | 2 (3.6) | 0 (0.0) | 4 (4.0) | 4 (4.0) | 0 (0.0) | 1 (2.4) | 1 (2.4) | 0 (0.0) | 0.054 | 0.094 | 0.578 |  |  |
| gamma glutamyl transferase | 10 (5.5) | 8 (4.4) | 2 (1.1) | 7 (5.3) | 4 (3.1) | 3 (2.3) | 5 (8.9) | 4 (7.1) | 1 (1.8) | 5 (5.1) | 5 (5.1) | 0 (0.0) | 1 (2.4) | 1 (2.4) | 0 (0.0) | 0.727 | 0.724 | 0.519 |  |  |
| lipase increased, other | 5 (2.8) | 4 (2.2) | 1 (0.6) | 1 (0.8) | 1 (0.8) | 0 (0.0) | 0 (0.0) | 0 (0.0) | 0 (0.0) | 1 (1.0) | 1 (1.0) | 0 (0.0) | 1 (2.4) | 1 (2.4) | 0 (0.0) | 0.478 | 0.643 | 0.771 |  |  |
| **Skin** | 12 (6.6) | 10 (5.5) | 2 (1.1) | 13 (9.9) | 11 (8.4) | 2 (1.5) | 7 (12.5) | 7 (12.5) | 0 (0.0) | 36 (36.4) | 36 (36.4) | 1 (1.0) | 11 (26.8) | 11 (26.8) | 0 (0.0) | <0.001 | <0.001 | 0.846 |  |  |
| vitiligo | 2 (1.1) | 2 (1.1) | 0 (0.0) | 0 (0.0) | 0 (0.0) | 0 (0.0) | 0 (0.0) | 0 (0.0) | 0 (0.0) | 21 (21.2) | 21 (21.2) | 0 (0.0) | 2 (4.9) | 2 (4.9) | 0 (0.0) | <0.001 | <0.001 |  |  |  |
| pruritus | 2 (1.1) | 2 (1.1) | 0 (0.0) | 6 (4.6) | 5 (3.8) | 1 (0.8) | 2 (3.6) | 2 (3.6) | 0 (0.0) | 3 (3.0) | 3 (3.0) | 0 (0.0) | 2 (4.9) | 2 (4.9) | 0 (0.0) | 0.409 | 0.517 | 0.578 |  |  |
| rash | 6 (3.3) | 6 (3.3) | 0 (0.0) | 6 (4.6) | 5 (3.8) | 1 (0.8) | 5 (8.9) | 5 (8.9) | 0 (0.0) | 5 (5.1) | 5 (5.1) | 0 (0.0) | 4 (9.8) | 4 (9.8) | 0 (0.0) | 0.31 | 0.257 | 0.578 |  |  |
| other | 3 (1.7) | 1 (0.6) | 2 (1.1) | 5 (3.8) | 4 (3.1) | 1 (0.8) | 0 (0.0) | 0 (0.0) | 0 (0.0) | 14 (14.1) | 13 (13.1) | 1 (1.0) | 3 (7.3) | 3 (7.3) | 0 (0.0) | <0.001 | <0.001 | 0.899 |  |  |
| **Hepatobiliary** | 4 (2.2) | 3 (1.7) | 1 (0.6) | 12 (9.2) | 10 (7.6) | 2 (1.5) | 4 (7.1) | 4 (7.1) | 0 (0.0) | 9 (9.1) | 7 (7.1) | 2 (2.0) | 7 (17.1) | 7 (17.1) | 1 (2.4) | 0.007 | 0.004 | 0.632 |  |  |
| **Gastrointestinal** | 6 (3.3) | 5 (2.8) | 1 (0.6) | 9 (6.9) | 8 (6.1) | 2 (1.5) | 2 (3.6) | 2 (3.6) | 0 (0.0) | 10 (10.1) | 7 (7.1) | 4 (4.0) | 9 (22.0) | 9 (22.0) | 0 (0.0) | <0.001 | <0.001 | 0.114 |  |  |
| **Hematologic** | 12 (6.6) | 10 (5.5) | 3 (1.7) | 6 (4.6) | 4 (3.1) | 2 (1.5) | 3 (5.4) | 2 (3.6) | 1 (1.8) | 2 (2.0) | 2 (2.0) | 0 (0.0) | 6 (14.6) | 3 (7.3) | 4 (9.8) | 0.057 | 0.481 | 0.004 | | |
| thrombocytopenia | 5 (2.8) | 5 (2.8) | 0 (0.0) | 5 (3.8) | 3 (2.3) | 2 (1.5) | 1 (1.8) | 0 (0.0) | 1 (1.8) | 0 (0.0) | 0 (0.0) | 0 (0.0) | 6 (14.6) | 2 (4.9) | 4 (9.8) | <0.001 | 0.233 | <0.001 | | |
| white blood cell count | 7 (3.9) | 4 (2.2) | 3 (1.7) | 1 (0.8) | 1 (0.8) | 0 (0.0) | 2 (3.6) | 2 (3.6) | 0 (0.0) | 0 (0.0) | 0 (0.0) | 0 (0.0) | 2 (4.9) | 0 (0.0) | 2 (4.9) | 0.13 | 0.265 | 0.039 | | |
| hemoglobin, other | 2 (1.1) | 2 (1.1) | 0 (0.0) | 1 (0.8) | 1 (0.8) | 0 (0.0) | 0 (0.0) | 0 (0.0) | 0 (0.0) | 2 (2.0) | 2 (2.0) | 0 (0.0) | 2 (4.9) | 1 (2.4) | 2 (4.9) | 0.263 | 0.731 | <0.001 | | |
| **Renal and urinary** | 7 (3.9) | 5 (2.8) | 2 (1.1) | 13 (9.9) | 12 (9.2) | 2 (1.5) | 4 (7.1) | 3 (5.4) | 1 (1.8) | 4 (4.0) | 3 (3.0) | 1 (1.0) | 0 (0.0) | 0 (0.0) | 0 (0.0) | 0.063 | 0.035 | 0.934 | | |
| **General** | 1 (0.6) | 1 (0.6) | 0 (0.0) | 3 (2.3) | 3 (2.3) | 0 (0.0) | 4 (7.1) | 4 (7.1) | 0 (0.0) | 7 (7.1) | 7 (7.1) | 0 (0.0) | 3 (7.3) | 3 (7.3) | 0 (0.0) | 0.012 | 0.012 |  | | |
| fever | 1 (0.6) | 1 (0.6) | 0 (0.0) | 1 (0.8) | 1 (0.8) | 0 (0.0) | 2 (3.6) | 2 (3.6) | 0 (0.0) | 5 (5.1) | 5 (5.1) | 0 (0.0) | 2 (4.9) | 2 (4.9) | 0 (0.0) | 0.055 | 0.055 |  | | |
| fatigue | 0 (0.0) | 0 (0.0) | 0 (0.0) | 1 (0.8) | 1 (0.8) | 0 (0.0) | 2 (3.6) | 2 (3.6) | 0 (0.0) | 3 (3.0) | 3 (3.0) | 0 (0.0) | 1 (2.4) | 1 (2.4) | 0 (0.0) | 0.125 | 0.125 |  | | |
| other | 0 (0.0) | 0 (0.0) | 0 (0.0) | 1 (0.8) | 1 (0.8) | 0 (0.0) | 0 (0.0) | 0 (0.0) | 0 (0.0) | 0 (0.0) | 0 (0.0) | 0 (0.0) | 0 (0.0) | 0 (0.0) | 0 (0.0) | 0.578 | 0.578 |  | | |
| **Respiratory** | 4 (2.2) | 1 (0.6) | 3 (1.7) | 3 (2.3) | 3 (2.3) | 0 (0.0) | 1 (1.8) | 0 (0.0) | 1 (1.8) | 7 (7.1) | 2 (2.0) | 5 (5.1) | 1 (2.4) | 0 (0.0) | 1 (2.4) | 0.182 | 0.441 | 0.105 | | |
| pneumonitis | 4 (2.2) | 1 (0.6) | 3 (1.7) | 1 (0.8) | 1 (0.8) | 0 (0.0) | 1 (1.8) | 0 (0.0) | 1 (1.8) | 6 (6.1) | 1 (1.0) | 5 (5.1) | 1 (2.4) | 0 (0.0) | 1 (2.4) | 0.148 | 0.918 | 0.105 | | |
| cough, other | 0 (0.0) | 0 (0.0) | 0 (0.0) | 2 (1.5) | 2 (1.5) | 0 (0.0) | 0 (0.0) | 0 (0.0) | 0 (0.0) | 1 (1.0) | 1 (1.0) | 0 (0.0) | 0 (0.0) | 0 (0.0) | 0 (0.0) | 0.419 | 0.419 |  | | |
| **Vascular** | 0 (0.0) | 0 (0.0) | 0 (0.0) | 6 (4.6) | 5 (3.8) | 1 (0.8) | 1 (1.8) | 1 (1.8) | 0 (0.0) | 2 (2.0) | 2 (2.0) | 0 (0.0) | 5 (12.2) | 4 (9.8) | 1 (2.4) | <0.001 | 0.004 | 0.188 |  |  |
| **Infections** | 1 (0.6) | 0 (0.0) | 1 (0.6) | 2 (1.5) | 1 (0.8) | 1 (0.8) | 1 (1.8) | 0 (0.0) | 1 (1.8) | 3 (3.0) | 0 (0.0) | 3 (3.0) | 1 (2.4) | 0 (0.0) | 1 (2.4) | 0.594 | 0.578 | 0.453 |  |  |
| **Musculoskeletal** | 1 (0.6) | 0 (0.0) | 1 (0.6) | 3 (2.3) | 3 (2.3) | 0 (0.0) | 2 (3.6) | 2 (3.6) | 0 (0.0) | 3 (3.0) | 3 (3.0) | 0 (0.0) | 0 (0.0) | 0 (0.0) | 0 (0.0) | 0.351 | 0.148 | 0.771 |  |  |
| **Cardiac** | 1 (0.6) | 1 (0.6) | 0 (0.0) | 0 (0.0) | 0 (0.0) | 0 (0.0) | 2 (3.6) | 1 (1.8) | 1 (1.8) | 2 (2.0) | 0 (0.0) | 2 (2.0) | 1 (2.4) | 1 (2.4) | 0 (0.0) | 0.194 | 0.275 | 0.141 |  |  |
| **Nervous system** | 2 (1.1) | 1 (0.6) | 1 (0.6) | 0 (0.0) | 0 (0.0) | 0 (0.0) | 0 (0.0) | 0 (0.0) | 0 (0.0) | 2 (2.0) | 2 (2.0) | 0 (0.0) | 1 (2.4) | 1 (2.4) | 0 (0.0) | 0.424 | 0.29 | 0.771 |  |  |
| **Ear and labyrinth** | 0 (0.0) | 0 (0.0) | 0 (0.0) | 0 (0.0) | 0 (0.0) | 0 (0.0) | 1 (1.8) | 1 (1.8) |  | 0 (0.0) | 0 (0.0) | 0 (0.0) | 0 (0.0) | 0 (0.0) | 0 (0.0) | 0.088 | 0.088 |  |  |  |
| **Eye** | 0 (0.0) | 0 (0.0) | 0 (0.0) | 0 (0.0) | 0 (0.0) | 0 (0.0) | 0 (0.0) | 0 (0.0) | 0 (0.0) | 1 (1.0) | 1 (1.0) | 0 (0.0) | 0 (0.0) | 0 (0.0) | 0 (0.0) | 0.387 | 0.387 |  |  |  |
| **Reproductive system** | 0 (0.0) | 0 (0.0) | 0 (0.0) | 1 (0.8) | 1 (0.8) | 0 (0.0) | 0 (0.0) | 0 (0.0) | 0 (0.0) | 0 (0.0) | 0 (0.0) | 0 (0.0) | 0 (0.0) | 0 (0.0) | 0 (0.0) | 0.578 | 0.578 |  |  |  |

Table S8. The impact of different anti-PD-1 based combination therapy on any grade irAEs, stratified by sex, age, biologic subtypes, number of metastases, anti-PD-1

| Variables | Subgroups | PD-1 (n=181) | | PD-1+TKI (n=131) | | PD-1+anti-VEGF (n=56) | | PD-1+IFN-α1b (n=99) | | PD-1+IFN-α1b+TKI (n=41) | | P value for interaction |
| --- | --- | --- | --- | --- | --- | --- | --- | --- | --- | --- | --- | --- |
|  |  | n | OR | n | OR (95% Cl); P | n | OR (95% Cl); P | n | OR (95% Cl); P | n | OR (95% Cl); P |  |
| Sex |  |  |  |  |  |  |  |  |  |  |  | 0.8278 |
|  | Male | 89 | 1 | 62 | 1.9 (1.0, 3.6) 0.0679 | 32 | 2.2 (1.0, 5.3) 0.0631 | 46 | 3.7 (1.6, 8.3) 0.0017 | 23 | 4.9 (1.5, 15.4) 0.0073 |  |
|  | Female | 92 | 1 | 69 | 2.1 (1.1, 4.0) 0.0327 | 24 | 1.7 (0.7, 4.3) 0.2807 | 53 | 4.7 (2.0, 11.1) 0.0004 | 18 | 4.2 (1.1, 15.5) 0.0312 |  |
| Age |  |  |  |  |  |  |  |  |  |  |  | 0.0627 |
|  | ≤65 | 120 | 1 | 82 | 1.4 (0.8, 2.4) 0.2813 | 35 | 2.1 (0.9, 4.7) 0.0667 | 70 | 5.8 (2.7, 12.4) <0.001 | 33 | 5.4 (2.0, 15.0) 0.0011 |  |
|  | >65 | 61 | 1 | 49 | 4.3 (1.8, 10.8) 0.002 | 21 | 1.7 (0.6, 4.8) 0.3179 | 29 | 2.2 (0.9, 5.8) 0.1012 | 8 | 2.5 (0.5, 13.6) 0.275 |  |
| Subtypes |  |  |  |  |  |  |  |  |  |  |  | 0.3740 |
|  | Cutaneous | 45 | 1 | 16 | 2.6 (0.7, 10.6) 0.1733 | 12 | 1.2 (0.3, 4.7) 0.7769 | 24 | 2.3 (0.7, 7.3) 0.1559 | 14 | 2.2 (0.5, 9.1) 0.2665 |  |
|  | Acral | 75 | 1 | 38 | 2.1 (0.9, 4.6) 0.0755 | 21 | 3.0 (1.1, 8.6) 0.0395 | 45 | 6.5 (2.6, 16.5) <0.0001 | 15 | 3.3 (1.0, 11.4) 0.0564 |  |
|  | Mucosal | 39 | 1 | 69 | 2.9 (1.3, 6.5) 0.0117 | 20 | 2.2 (0.7, 6.6) 0.1737 | 21 | 5.0 (1.4, 17.4) 0.0126 | 9 | inf. (0.0, Inf) 0.9893 |  |
| No.of metastases |  |  |  |  |  |  |  |  |  |  |  | 0.9208 |
|  | <3 | 130 | 1 | 95 | 1.7 (1.0, 2.9) 0.0630 | 36 | 2.3 (1.0, 5.4) 0.0445 | 71 | 4.8 (2.2, 10.1) <0.0001 | 26 | 4.3 (1.4, 13.2) 0.0108 |  |
|  | ≥3 | 51 | 1 | 36 | 2.9 (1.2, 7.0) 0.0207 | 20 | 1.7 (0.6, 5.0) 0.2948 | 28 | 3.6 (1.3, 9.6) 0.0119 | 15 | 5.7 (1.4, 22.8) 0.0135 |  |
| PD-1 |  |  |  |  |  |  |  |  |  |  |  | 0.8531 |
|  | Toripalimab | 158 | 1 | 107 | 1.8 (1.1, 3.0) 0.0200 | 52 | 1.9 (1.0, 3.7) 0.0543 | 79 | 3.6 (1.9, 6.9) <0.0001 | 29 | 5.8 (1.9, 17.4) 0.0018 |  |
|  | Pembrolizumab | 23 | 1 | 24 | 2.7 (0.8, 9.4) 0.1081 | 4 | 2.7 (0.2, 30.5) 0.4100 | 20 | 8.2 (1.5, 44.0) 0.0135 | 12 | 2.8 (0.6, 12.8) 0.1984 |  |

Adjusted for age, gender, ECOG PS, stage, biologic subtypes, number of metastases, brain metastases, Liver/kidney dysfunction, autoimmune disease, prior ICIs, prior cytokine, prior TKI, line of therapy, whether chemotherapy was added, anti-PD-1, duration of anti-PD-1, except the stratification factor itself.

Table S9. The impact of different anti-PD-1 based combination therapy on grade 3-5 irAEs, stratified by sex, age, biologic subtypes, number of metastases, anti-PD-1

| Variables | Subgroups | PD-1 (n=181) | | PD-1+TKI (n=131) | | PD-1+anti-VEGF (n=56) | | PD-1+IFN-α1b (n=99) | | PD-1+IFN-α1b+TKI (n=41) | | P value for interaction |
| --- | --- | --- | --- | --- | --- | --- | --- | --- | --- | --- | --- | --- |
|  |  | n | OR | n | OR （95% Cl) |  | OR （95% Cl) |  | OR （95% Cl) |  | OR （95% Cl) |  |
| Sex |  |  |  |  |  |  |  |  |  |  |  |  |
|  | Male | 89 | 1 | 62 | 2.3 (0.8, 6.3) 0.1210 | 32 | 1.7 (0.5, 6.1) 0.4380 | 46 | 2.5 (0.8, 7.3) 0.1029 | 23 | 3.3 (0.9, 11.4) 0.0656 | 0.8560 |
|  | Female | 92 | 1 | 69 | 1.1 (0.4, 2.9) 0.8852 | 24 | 0.7 (0.2, 3.7) 0.7171 | 53 | 1.9 (0.7, 4.9) 0.1834 | 18 | 4.1 (1.3, 13.3) 0.0191 |  |
| Age |  |  |  |  |  |  |  |  |  |  |  | 0.5741 |
|  | ≤65 | 120 | 1 | 82 | 0.8 (0.3, 2.0) 0.6690 | 35 | 1.6 (0.6, 4.4 ) 0.3980 | 70 | 2.1 (0.9, 4.6) 0.3980 | 33 | 3.3 (1.3, 8.3) 0.0119 |  |
|  | >65 | 61 | 1 | 49 | 5.0 (1.3, 19.2) 0.0204 | 21 | 0.0 (0.0, Inf) 0.9913 | 29 | 2.2 (0.4, 11.8) 0.3452 | 8 | 2.8 (0.3, 30.3) 0.4058 |  |
| Subtypes |  |  |  |  |  |  |  |  |  |  |  | 0.9356 |
|  | Cutaneous | 45 | 1 | 16 | 0.5 (0.1, 4.9) 0.5802 | 12 | 1.6 (0.3, 9.5) 0.6048 | 24 | 0.7 (0.1, 4.1) 0.7168 | 14 | 6.0 (1.5, 24.5) 0.0127 |  |
|  | Acral | 75 | 1 | 38 | 2.1 (0.5, 8.9) 0.3179 | 21 | 0.0 (0.0, Inf) 0.9912 | 45 | 3.8 (1.1, 13.6) 0.0371 | 15 | 2.7 (0.5, 16.5) 0.2733 |  |
|  | Mucosal | 39 | 1 | 69 | 4.3 (0.9, 20.1) 0.0646 | 20 | 4.6 (0.8, 27.9) 0.0946 | 21 | 9.2 (1.7, 50.0) 0.0098 | 9 | 5.3 (0.6, 44.0) 0.1237 |  |
| No.of metastases |  |  |  |  |  |  |  |  |  |  |  | 0.1715 |
|  | <3 | 130 | 1 | 95 | 1.7 (0.7, 4.2) 0.2222 | 36 | 1.5 (0.4, 5.1) 0.5159 | 71 | 3.2 (1.4, 7.6) 0.0078 | 26 | 5.3 (1.9, 15.3) 0.0018 |  |
|  | ≥3 | 51 | 1 | 36 | 1.3 (0.4, 4.1) 0.7051 | 20 | 0.7 (0.1, 3.7) 0.6725 | 28 | 0.8 (0.2, 3.2) 0.7009 | 15 | 1.6 (0.4, 7.0) 0.5536 |  |
| Anti-PD-1 |  |  |  |  |  |  |  |  |  |  |  | 0.9991 |
|  | Toripalimab | 158 | 1 | 107 | 2.0 (0.9, 4.4) 0.0944 | 52 | 1.3 (0.4, 3.9) 0.6439 | 79 | 2.0 (0.8, 4.7) 0.1260 | 29 | 3.9 (1.4, 10.9) 0.0103 |  |
|  | Pembrolizumab | 23 | 1 | 24 | 0.5 (0.1, 2.5) 0.4046 | 4 | 1.2 (0.1, 14.2) 0.8850 | 20 | 1.9 (0.5, 7.5) 0.3370 | 12 | 1.8 (0.4, 8.5) 0.4592 |  |

Adjusted for age, gender, ECOG PS, stage, biologic subtypes, number of metastases, brain metastases, Liver/kidney dysfunction, autoimmune disease, prior ICIs, prior cytokine, prior TKI, line of therapy, whether chemotherapy was added, anti-PD-1, duration of anti-PD-1, except the stratification factor itself.
